# Synthesis of some new isoxazole compounds and their biological tyrosinase and antioxidant activities

**DOI:** 10.55730/1300-0527.3364

**Published:** 2021-12-31

**Authors:** Seda FANDAKLI

**Affiliations:** Department of Nutrition and Dietetics, Faculty of Health Sciences, Avrasya University, Trabzon, Turkey

**Keywords:** Isoxazole, tyrosinase inhibition, antioxidant activity

## Abstract

In this study, 7 new isoxazole compounds (**8–14**) were synthesized from the cyclization of chalcone compounds (**1–7**) containing different functional groups with hydroxylamine hydrochloride in alkaline medium. Tyrosinase and antioxidant properties of **8–14** were investigated. IC_50_ values for the tyrosinase enzyme inhibition of compounds **8**, **11**, **12**, and **13** were varied between 61.47 ± 3.46 and 188.52 ± 5.85, while compounds **9**, **10** and **14** did not show any inhibition effect. The antioxidant properties of **8–14** were investigated by DPPH and CUPRAC methods. Compound **12** showed the best DPPH radical scavenging activity (SC_50_: 40.21 ± 2.71), while **12** and **13** had shown the greatest Cupric ion reducing effect as 1.233 ± 0.015 and 1.245 ± 0.019 mg TEAC/mg, respectively. As a result, the change of functional groups in the synthesized compounds caused a significant difference in the biological properties of **8–14**.

## 1. Introduction

The isoxazole and its derivatives are an important intermediate for the synthesis of new chemicals in medicinal chemistry, which have been increased in last decades. Isoxazole nucleus had shown a wide spectrum of biological activity. Easy synthesis of isoxazole ring has been as an object of interest for the scientist from research group all over the world. The synthesis of isoxazole compounds have resulted in multiple corresponding antibacterial drugs in the market (Sulfisoxazole, Flucloxacillin, Dicloxacillin, Leflunomide, Risperidone, Oxacillin, Sulfamethoxazole, and Cloxacillin) and there are many patent related with isoxazole [1]. Leflunomide has been approved for the management of the signs and symptoms of active rheumatoid arthritis (RA) to improve physical function. Sulfisoxazole has been approved for the treatment of severe, repeated, or long-lasting urinary tract infections by inhibiting the enzyme dihydropteroate synthetase. Risperidone has been approved for the treatment of schizophrenia in adults [1]. 1,3-Dipolar cycloaddition of α, β-unsaturated ketone or 1,3-diketone is a widely used method for the synthesis of isoxazole [2]. One of the substituted isoxazole synthesis described using oxime of a ketone [3]. Although there are many methods were reported for the synthesis or modification of the isoxazole ring [1–9]. Isoxazoles play a fundamental role in the synthesis of numerous biologically active drugs such as antimicrobial, antiviral, anticancer, immunomodulatory, antiinflammatory, antiplatelet, antithrombotic, antitriglyceride, antidiabetic, analgesic, anticonvulsant, and anti-Alzheimer agents [2], pesticides and insecticides [10–11]. Thus, the synthesis of novel isoxazole derivatives remains a main focus of medicinal research. In this work, the synthesis and biological activities of a new series of isoxazole analogues with a substituted two aromatic rings still needed and are reported here.

## 2. Experimental

### 2.1. Material and methods

Solvents (*n*-hexane, ethyl alcohol, and diethyl ether), aldehydes and ketone compounds (2/3/4-methoxyacetophenone, 3/4-hydroxyacetophenone, 2,4-dimethoxy-benzaldehyde, 2,5-dimethoxybenzaldehyde, 2,3,4-trimethoxybenzaldehyde, benzaldehyde, pyridine-2-carbaldeyde, and pyridine-4-carbaldeyde), 1,1-diphenyl-2-picrylhydrazil (DPPH), kojic acid and any used reagent were purchased from by Sigma-Aldrich, Fluka or Merck unless otherwise stated.

^1^H and ^13^C NMR spectra were obtained on a Bruker 400 MHz NMR spectrometer (400 MHz for ^1^H, 100 MHz for ^13^C), using TMS as an internal standard. CDCl_3_ and DMSO-d_6_ were used as NMR solvents. ^13^C and APT spectra were adjusted according to deutero solvent peaks. Chemical shifts were expressed in δ (ppm), and coupling constants (*J*) were reported in hertz (Hz). ACD NMR program was used for the interpretation of spectra. FT-IR spectra were taken using the Perkin-Elmer 1600 (ATR) (4000–400 cm^−1^) spectrophotometer. Melting points were determined using the Thermo-var apparatus fitted with a microscope. Normal phase silica gel (230–400 mesh) was used in vacuum column chromatography (VLC). TLC was carried out on Silica gel 60 F_254_, and the spots were visualized by UV lamp (254 nm and 366 nm) or spraying with 20% H_2_SO_4_ and heating.

### 2.1. General procedure for the synthesis of compounds 1–14

The synthesis of known chalcone compounds (**1–7**) was carried out using the Claisen-Schmidt condensation [12,13]. A mixture of chalcone (**1–7**) (10 mmol) and hydroxylamine hydrochloride (15 mmol) in ethyl alcohol (30 mL) was refluxed for 12 h in presence of 40% KOH (5 mL) [1–9]. The reaction mixture was controlled by TLC then cooled, poured into crushed ice and extracted with diethyl ether (30 mL × 3). Solvent was evaporated to give crude products which were chromatographed by column chromatography using increased polarity of *n*-hexane then ether mixture to give compounds **8–14** in the range of 45%–63% yield, respectively.

Compound **8** (3-(2-methoxyphenyl)-5-(2,3,4-trimethoxyphenyl)isoxazole): Yield: 63%, m.p. = 102–103 ^o^C, FT-IR (cm^−1^):1589 (C=C, aromatic ring),1606 (C=N), 2940 (=CH), ^1^H-NMR (δ, ppm, DMSO-d_6_): 3.97, 3.98 (s, 12 H, −OCH_3_), ar-H:[6.80–6.87 (m,1H), 7.04–7.15 (m, 2H), 7.42–7.50 (m,1H), 7.69–7.75 (m,1H), 7.91–7.97 (m,1H)], 7.25 (s,1H, isoxazole-H_4_), APT-NMR (100 MHz, DMSO-d_6_): 61.04, 60.67, 56.11, 55.61 (4× −OCH_3_), 103.35 (isoxazole-C_4_), ar-C [107.78 (CH), 115.25 (C),115.51 (CH), 118.54 (C), 120.93 (CH), 122.53 (CH), 129.65 (CH), 131.04 (CH), 142.65 (C), 151.27(C), 155.10 (C), 157.32 (C), 160.93 (isoxazole-C_3_), 165.35 (isoxazole-C_5_), ]. LC-TOF-MS: (m/z, %) [M+Na]^+^: 364.1159 (100), calc. 364,1187. Supplementary data are in Figures S1–S4.

Compound **9** (3-(4-methoxyphenyl)-5-(2,4-dimethoxyphenyl)isoxazole): Yield: 45%, m.p. = 93–94 ^o^C, FT-IR (cm^−1^):1594 (C=C, aromatic ring),1613 (C=N), 3058, 3006, 2987 (=CH), ^1^H-NMR (δ, ppm, DMSO-d_6_): 3.90 (s, 6H, −OCH_3_), 4.00 (s, 3H, −OCH_3_), ar-H:[ 6.60–6.66 (m, 2H), 6.94–6.98 (m, 2H), 7.82–7.98 (m, 3H)], 7.03 (s,1H, isoxazole-H_4_), APT-NMR (100 MHz, DMSO-d_6_): 55.39, 55.56, 55,64 (3 × −OCH_3_), 98.92 (isoxazole-C_4_), ar-C [99.66 (CH), 106.12 (CH), 109.95(C), 114.21 (2CH), 122.26 (C), 128.24 (2CH), 128.84 (CH), 157.61(C), 160.80 (C), 162.26 (C)], 162.58 (isoxazole-C_3_), 166.26 (isoxazole-C_5_),. LC-TOF-MS: (m/z, %) [M+H]^+^: 312.1235 (100), calc. 312.1222, [M+Na]^+^: 334.1059 (70), calc. 334.1049. Supplementary data are in Figures S5–S8.

Compound **10** (3-(3-hydroxyphenyl)-5-(phenyl)isoxazole): Yield: 62%, m.p. = 230–231 ^o^C, FT-IR (cm^−1^):1605 (C=N), 2962, 2836 (=CH), 3193, 3484 (−OH), ^1^H-NMR (δ, ppm, DMSO-d_6_): ar-H: [6.94 (d, 1H, *J*=6.8 Hz), 7.35–7.38 (m, 2H), 7.54 (d, 4H, *J*=8.0 Hz), 7.93 (d, 2H, *J*=6.4 Hz )], 7.31(s,1H, isoxazole-H_4_), 9.21 (phenolic,−OH) (s, 1H), APT-NMR (100 MHz, DMSO-d_6_): 99.00 (isoxazole-C_4_), ar-C [112.50 (CH), 116.92 (CH), 118.07 (CH), 127.06 (2 CH), 128.42 (C), 129,61 (C), 129.61 (2 CH), 130.76 (CH), 130.96 (CH), 158.40 (C)], 163.03 (isoxazole-C_3_), 170.29 (isoxazole-C_5_). LC-TOF-MS: (m/z, %) [M+H]^+^: 238.0856 (100), calc. 238.0844. Supplementary data are in Figures S9–S12.

Compound **11** (3-(3-hydroxyphenyl)-5-(2,4-dimethoxyphenyl)isoxazole): Yield: 60%, m.p. = 168–169 ^o^C, FT-IR (cm^−1^): 1572(C=C, aromatic ring), 1608 (C=N), 2940, 3010, 3007 (=CH), 3256 (−OH), ^1^H-NMR (δ, ppm, DMSO-d_6_): 3,85 (s, 3H, −OCH_3_), 3.98 (s, 3H, −OCH_3_) ar-H:[ 6.71 (d, 1H, *J*=8.8 Hz), 6.90 (d, 1H, *J*=7.2 Hz), 7.14 (s,1H), 7.30–7.36 (m,3H), 7.81 (d, 1H, *J*=8.8 Hz)], 6.75 (s,1H, isoxazole-H_4_), 9.76 (phenolic,−OH) (s, 1H), APT-NMR (100 MHz, DMSO): 56.33, 55.99 (2 × −OCH_3_), 99.24 (isoxazole-C_4_), ar-C [100.22 (CH), 106.48 (CH), 108.99 (C), 113.49 (CH), 117.59 (CH), 118.00 (CH), 128.56 (CH), 130.49 (C),130.63 (CH), 157.99 (C), 158.27(C), 162.79 (C)], 162.82 (isoxazole-C_3_), 166.56 (isoxazole-C_5_). LLC-TOF-MS: (m/z, %) [M+H]^+^: 298.1074, calc. 298.1080. Supplementary data are in Figures S13–S16.

Compound **12** (3-(4-hydroxyphenyl)-5-(2,5-dimethoxyphenyl)isoxazole): Yield: 65%, m.p. = 176–178 ^o^C,, FT-IR (cm^−1^):1575(C=C, aromatic ring), 1603 (C=N), 3061 (=CH), 3479 (−OH), ^1^H-NMR (δ, ppm, DMSO-d_6_): 3.76 (s, 3H,−OCH_3_), 3.84 (s, 3H,−OCH_3_), ar-H:[ 6.95 (d, 2H, *J*=8.8 Hz), 7.04–7.07 (m, 1H),7.33 (d, 1H, *J*=3.2 Hz), 7.16 (s, 1H), 7.76 (d, 2H, *J*=8.4 Hz)], 10.1 (phenolic,−OH) (s, 1H), 7.11 (m,1H, isoxazole −H_4_), APT-NMR (100 MHz, DMSO-d_6_): 55.94 (−OCH_3_), 56.60 (−OCH_3_), 99.86 (isoxazole-C_4_), ar-C [114.00 (CH), 114.06 (CH), 116.43 (2CH), 117.10 (CH), 127.85 (2CH),118.39 (C), 118.66 (C), 151.69 (C), 153.61(C), 159.84 (C)], 160.45 (isoxazole-C_3_), 169.67 (isoxazole-C_5_), LC-TOF-MS: (m/z, %) [M+H]^+^: 298.1065 (100), calc. 298.1080. Supplementary data are in Figures S17–S20.

Compound **13** (3-(3-methoxyphenyl)-5-(2-pyridinyl)isoxazole): Yield: 58%, m.p. = 98 ^o^C, FT-IR (cm^−1^): 1568 (C=C, aromatic ring), 1582 (C=N), 2962, 3049, 3084 (C=C), ^1^H-NMR (δ, ppm, DMSO-d_6_): 3.35 (s, 3H,−OCH_3_), ar-H:[ 7.10 (d, 1H, *J*=7.6 Hz), 7.45–7.56 (m, 4H), 8.0 (d, 1H, *J*=7.6 Hz), 8.07 (d, 1H, *J*=7.6 Hz), 8.76 (s,1H)], 7.65 (s,1H,isoxazole-H_4_), APT-NMR (100 MHz, DMSO-d_6_): 55.89 (−OCH_3_), 99.89 (isoxazole-C_4_), ar-C [111.22 (CH), 117.13 (CH), 118.40 (CH), 121.86 (CH), 125.66 (CH), 128.39 (C), 130.98 (CH), 138.04 (CH), 148.06 (C), 150.40 (CH), 160.23(C)], 164.06 (isoxazole-C_3_), 170.36 (isoxazole-C_5_), LC-TOF-MS: (m/z, %) [M+H]^+^: 253.0990 (100), calc. 253.0890. Supplementary data are in Figures S21–S24.

Compound **14** (3-(4-methoxyphenyl)-5-(4-pyridinyl)isoxazole): Yield: 55%, m.p. = 141–143 ^o^C, FT-IR (cm^−1^): 1573 (C=C, aromatic ring), 1610 (C=N), 2967, 3019, 3112 (=CH), ^1^H-NMR (δ, ppm, DMSO-d_6_): 3.83(s, 3H,−OCH_3_), ar-H:[7.09–7.11 (d, 2H, *J*=7.6 Hz), 7.78 (d, 2H, *J*=7.6 Hz), 7.82 (d, 2H, *J*=6.4 Hz), 8.78 (d, 2H, *J*=6.4 Hz)], 7.80 (s,1H, isoxazole-H_4_), APT-NMR (100 MHz, DMSO-d_6_): 55 (−OCH_3_), 102 (isoxazole-C_4_), ar-C [116 (2CH), 119 (2CH), 121 (C), 128 (2CH), 134 (C), 152 (2CH), 162 (C)], 164 (isoxazole-C_3_), 168 (isoxazole-C_5_). LC-TOF-MS: (m/z, %) [M+H]^+^: 253.0977 (100), calc., 253.0890, [M+Na]^+^: 275.0805 (60), calc. 275.0825. Supplementary data are in Figures S25–S28.

### 2.3. Biological activities

#### 2.3.1. Antioxidant activity

##### 2.3.1.1. Cupric ion reducing (CUPRAC) method

The capacity of **8–14** compounds to reduce cupric ions was established as it was described before [14]. A reaction mixture was prepared by mixing a volume of CuCl_2_ (0.250 mL, 10 mM), neocuproine (0.250 mL, 7.5 mM in ethanol) and NH_4_Ac buffer (0.250 mL, 1 M, pH 7.0) with standard/compound solutions at different concentrations. SA blank was prepared by adding sample solution to premixed reaction mixture CuCl_2_. The absorbances of the blank and sample were read at 450 nm after a 30-min incubation at room temperature. The absorbance of the blank was subtracted from that of the sample. CUPRAC activity was expressed as equivalents of trolox (mg TEAC/mg compound).

##### 2.3.1.2. DPPH free radical scavenging method

The DPPH free radical scavenging activities of **8–14** compounds were determined using the method described before [15]. Alcoholic solution of DPPH (1 mL, 0.1 M in methanol) was mixed with **8–14** compound solutions prepared in DMSO. The absorbance was noted at 517 nm after incubation in the dark for 50 min. DPPH gives a purple color in methanol. This color is decayed by antioxidant. So reduction in the absorbance indicates DPPH• scavenging activity. Trolox was used as standard radical scavenger and values were expressed as SC50 (μg compound per mL), the concentration of the samples that causes 50% scavenging of DPPH radical.

#### 2.3.2. Tyrosinase inhibition

The mushroom tyrosinase inhibition assay was carried out according previously reported method. A total of 100 μL of 100 mM phosphate buffer (pH 6.8), 20 μL of 250 U/mL mushroom and compounds **8–14** (5 μL) were prepared in 96 well plate [16,17]. After 10 min of a preincubation period at room temperature, the enzymatic reaction was initiated with the addition of l-DOPA (3 mM, 20 μL) as substrate. The absorbance was read at 475 nm. The compound concentration giving 50% inhibition of tyrosinase activity was determined by interpolation of concentration-response curves. Kojic acid was used as a positive control. The extract concentration giving 50% (IC_50_) of the original tyrosinase activity was determined.

## 3. Results

### 3.1. Synthesis

The synthetic procedure of the target compounds was shown in Figure. Commercially available different aromatic ketone with aldehydes using NaOH were yielded known chalcones (**1–7**) [18–24] through the Claisen–Schmidt condensation [12,13] which then were condensed with hydroxylamine hydrochloride in alkaline medium to give their corresponding isoxazole by intermolecular cycloaddition reaction (Figure) [25]. All new compounds (**8–14**) were characterized by ^1^H NMR, ^13^C/APT NMR, FT-IR, and LC-TOF/ MS (Supplementary data are in Figures S1–S28). Then these compounds were screened for their tyrosinase enzyme inhibition and antioxidant properties.

Isoxazole is an azole with an oxygen atom next to the nitrogen. Isoxazole rings are found in some natural products and a number of drugs such as ibotenic acid, COX-2 inhibitor valdecoxib, furoxan, cloxacillin, dicloxacillin, and flucloxacillin. The synthetic androgenic steroid danazol also has an isoxazole ring. The synthesis of substituted isoxazoles are well developed in literature to possess significant biological activities. In the literature, Kang et al. [26] described the synthesis of a series of new isoxazole derivatives showed potent antibacterial properties against different gram-positive and gram-negative organisms. Lamani et al. [27] reported the synthesis of a new series of benzisoxazolyl imidazothiadiazoles and evaluated their antibacterial potential. Yamuna et al. [28] also described a synthesis and biological properties of isoxazolo pyrimidocyclohepta[b]indoles, the best activity against *Mycobacterium tuberculosis* strain H37Rv were reported for an isoxazole derivative with MIC value 3.12 mg/mL. Another group of isoxazole derivatives is a series of (1,4-phenylene)bis(arylsulfonylisoxazoles) synthesized by Lavanya et al. [29] by 1,3-dipolar cycloaddition. Sharp et al. [30] described the properties of another isoxazole Hsp90 inhibitor. Kamal et al. [31] also reported the synthesis of 3,5-diaryl isoxazole linked with quinazolinone and assayed for their anticancer activity. There are many manuscript related with the synthesis and biological evaluation of isoxazoles [1–9].

### 3.2. Biological activities

#### 3.2.1. Tyrosinase activity of compounds 8–14

The activities on tyrosinase of synthesized compounds **8–14** were performed according to a modified method with Kojic acid as positive control [17, 32]. The activity effects of **8–14** were summarized in Table 1. As shown in Table 1, among the synthesis compounds **8**, and **11–13** showed good to moderate activating effect on tyrosinase. The potencies of compound **8** (with the value of IC_50_ = 66.74 ± 3.96 μg/mL), **11** (IC_50_ = 61.47 ± 3.46 μg/mL), **12** (IC_50_ = 91.41 ± 4.15 μg/mL) and **13** (IC_50_ = 188.52 ± 5.85 μg/mL) were lower to the positive control kojic acid, of which IC_50_ was 3.2 ± 0.29 μg/mL. The position, number and nature of the substituent on benzene and pyridine rings were varied in order to identify the most appropriate group. The compounds with 3-hydroxy and 2,4-dimethoxy (**11**) showed higher activity compared to with 2-methoxy and 2,3,4-trimethoxy (**8**), 4-hydroxy and 2,5-dimethoxy (**12**), and 3-methoxy and 4-pyridinyl (**13**), which suggested that the strong electron donating group and the number of methoxy may be favorable to enhance the activity. However, the activity dropped rapidly when the less methoxy substituted and the benzene on isoxazole was replaced by pyridine, which suggested that benzene was fundamental for the activity [25].

Synthetic and natural tyrosinase inhibitors are promising therapeutic agents for skincare and cosmetic products. Tyrosinase is the key enzyme in the melanogenesis pathway, responsible for the formation of melanin pigment. The most common target for inhibiting the melanogenesis pathway is the direct inhibition of the catalytic activity of tyrosinase, and the most widely used hypopigmented agents are tyrosinase inhibitors [17]. Tyrosinase inhibitors, which can inhibit melanin biosynthesis, are currently used in various hyperpigmentation and cosmetic products to control freckle formation [32]. Kojic acid is a fungal metabolite that exhibits inhibitory activity against tyrosinase. Kojic acid is often used as a positive control to discover new tyrosinase inhibitors [33,34].

In the literature, a series of novel isoxazole chalcone derivatives have been synthesized, and evaluated for tyrosinase activities. Among them, 1-(4-((3-phenylisoxazol-5-yl) methoxy) phenyl) -3-phenylprop-2-en-1-one was reported as a potent tyrosinase activator [35]. In another study, isoxazole chalcone was reported that they inhibit the monopenolase activity of fungal tyrosinase [25]. In another work, novel 2- (3, 5-disubstituted isoxazolyl) -1, 5-benzodiazepines has been reported and those hybrid compounds exhibited moderate to significant antityrosinase activities [36]. The tyrosinase inhibitory activity of the natural curcuminoid analogs on both L-tyrosine and DOPA substrates were reported. The isoxazole analog showed IC_50_ values of 8.3, μM, and were 20.9-fold more active than kojic acid [37]. Thus, they showed awareness of tyrosinase activities according to the substitution difference of isoxazole compounds.

#### 3.2.2. Antioxidant activity of compounds 8–14

Antioxidant properties of synthesized compounds **8–14** were made according to CUPRAC and DPPH methods [14, 38–39] as seen in Table 2. The highest CUPRAC values of isoxazole compounds (**8–14**) were 1.245 ± 0.019, 1.233.33 ± 0.015, and 1.189.33 ± 0.021 (μg/mL trolox/gram DW) in compounds **13**, **12**, and **14**, and the lowest DPPH values for compounds **11** and **10** were found to be 0.584 ± 0.011 mg/mL and 0.789 ± 0.009 mg/mL, respectively. When the activities of all compounds according to CUPRAC and DPPH were examined, it was seen that compound **13** for CUPRAC, and compound **11** for DPPH were the most effective antioxidant compounds. When looking at the substitution positions of these compounds, it has been found that they are generally more effective when they are substituted at the 3-position phenyl ring which is substituted at the C-3 position of isoxazole. Isoxazole having 3-hydroxy and 3-methoxy at the phenyl group which are attached at the C-3 position of isoxazoles (**11** and **13**) showed the greatest activity due to the electron donation and formation of phenoxy radical.

Cancer is one of the leading causes of mortality in the world. There has been a lot of effort to discover new anticarcinogenic agents that allow treatment with fewer side effects. Isoxazole is the anticancer agent. A series of isoxazole-carboxamide derivatives were reported and evaluated for their antioxidant activity in DPPH assay. One of the compound was reported the most active as antioxidant agent (IC_50_ = 7.8 ± 1.21 μg/mL) compared with Trolox (IC_50_ 2.75 μg/mL) [40]. Antioxidant activity of isoxazole-based chalcones were reported [41]. A novel series of indolyl isoxazole derivatives were synthesized and their antioxidant activity were evaluated [42]. The substituted isoxazole derivatives displayed antioxidant activity [43]. The disubstituted and trisubstituted isoxazoles have been reported to exhibit antioxidant activity [44]. Antioxidant activity of novel functionalized isoxazole derivatives were reported and among them 5-amino-3(pyridine-4-yl) isoxazole-4-carbonitrile was highly significant [45]. As seen in the literature, compounds having isoxazole ring with different substituents had shown very different biological activities. In this study, tyrosinase and antioxidant activities of 7 different isoxazole compounds, which are not found in the literature, were reported.

## 4. Conclusion

In summary, this article is based on synthesized new isoxazole derivatives which display wide spectrum of biological potential such as anticancer, antiinflammatory, antimicrobial, antiviral, antiplatelet, immunomodulatory, antithrombotic, antitriglyceride, antidiabetic, analgesic, anticonvulsant, anti-Alzheimer agents, pesticides and insecticides. The heterocyclic moiety being so versatile in nature offers the medicinal chemist to explore more about the data mentioned in this article, which will be a great help to prospective researches working in this area for further studies. Thus, the synthesis of new isoxazole is still needed. Compounds **11** and **8** could be used as new potential tyrosinase inhibitor and **13** as antioxidant agent. It may be concluded that isoxazole moiety is an important heterocyclic compound as an essential constituent of large number of marketed drugs. Isoxazole compounds have a great potential to be explored for the development of new chemical compounds to treat a variety of clinically important diseases.

## Supplementary Material

**Table:** 

Table of Contents	Page
Figure S1. ^1^H-NMR spectrum of compound **8** (CDCl_3_, 400 MHz) ...........................................	2
Figure S2. APT-NMR spectrum of compound **8** (CDCl_3_,100 MHz) .........................................	2
Figure S3. LC-Q-TOF/MS spectrum of compound **8** ................................................................	3
Figure S4. ATR (FT-IR) spektrum of compounds **8** ..................................................................	3
Figure S5. ^1^H-NMR spectrum of compound **9** (CDCl_3_, 400 MHz) ...........................................	4
Figure S6. APT-NMR spectrum of compound **9** (CDCl_3_,100 MHz) .........................................	4
Figure S7. LC-Q-TOF/MS spectrum of compound **9** ................................................................	5
Figure S8. ATR (FT-IR) spektrum of compounds **9** ..................................................................	5
Figure S9. ^1^H-NMR spectrum of compound **10** (DMSO, 400 MHz) .......................................	6
Figure S10. APT-NMR spectrum of compound **10** (DMSO,100 MHz) ....................................	6
Figure S11. LC-Q-TOF/MS spectrum of compound **10** ............................................................	7
Figure S12. ATR (FT-IR) spektrum of compound **10** ...............................................................	7
Figure S13. ^1^H-NMR spectrum of compound **11** (DMSO, 400 MHz) ......................................	8
Figure S14. APT-NMR spectrum of compound **11** (DMSO,100 MHz) ....................................	8
Figure S15. LC-Q-TOF/MS spectrum of compound **11** ............................................................	9
Figure S16. ATR (FT-IR) spektrum of compound **11** ...............................................................	9
Figure S17. ^1^H-NMR spectrum of compound **12** (DMSO, 400 MHz) ....................................	10
Figure S18. APT-NMR spectrum of compound **12** (DMSO,100 MHz) ..................................	10
Figure S19. LC-Q-TOF/MS spectrum of compound **12** ..........................................................	11
Figure S20. ATR (FT-IR) spektrum of compound **12** .............................................................	11
Figure S21. ^1^H-NMR spectrum of compound **13** (DMSO, 400 MHz) ....................................	12
Figure S22. ^13^C-NMR spectrum of compound **13** (DMSO, 100 MHz) ...................................	12
Figure S23. LC-Q-TOF/MS spectrum of compound **13** ..........................................................	13
Figure S24. ATR (FT-IR) spektrum of compound **13** .............................................................	13
Figure S25. ^1^H-NMR spectrum of compound **14** (DMSO, 400 MHz) ....................................	14
Figure S26. APT-NMR spectrum of compound **14** (DMSO, 100 MHz) ................................	14
Figure S27. LC-Q-TOF/MS spectrum of compound **14** .........................................................	15
Figure S28. ATR (FT-IR) spektrum of compound **14** .............................................................	15

Figure S1 ^1^H-NMR spectrum of compound **8** (CDCl_3_, 400 MHz)

Figure S2 APT-NMR spectrum of compound **8** (CDCl_3_, 100 MHz)

Figure S3 LC-Q-TOF/MS spectrum of compound 8

Figure S4 ATR (FT-IR) spectrum of compound 8

Figure S5 ^1^H-NMR spectrum of compound 9 (CDCl_3_, 400 MHz)

Figure S6 APT-NMR spectrum of compound 9 (CDCl_3_, 100 MHz)

Figure S7 LC-Q-TOF/MS spectrum of compound 9

Figure S8 ATR (FT-IR) spektrum of compound 9

Figure S9 ^1^H-NMR spectrum of compound 10 (DMSO-d_6_, 400 MHz)

Figure S10 APT-NMR spectrum of compound 10 (DMSO-d_6_, 100 MHz)

Figure S11 LC-Q-TOF/MS spectrum of compound 10

Figure S12 ATR (FT-IR) spektrum of compound 10

Figure S13 ^1^H-NMR spectrum of compound 11 (DMSO-d_6_, 400 MHz)

Figure S14 APT-NMR spectrum of compound 11 (DMSO-d_6_, 100 MHz)

Figure S15 LC-Q-TOF/MS spectrum of compound 11

Figure S16 ATR (FT-IR) spectrum of compound 11

Figure S17 ^1^H-NMR spectrum of compound 12 (DMSO-d_6_, 400 MHz)

Figure S18 APT-NMR spectrum of compound 12 (DMSO-d_6_, 100 MHz)

Figure S19 LC-Q-TOF/MS spectrum of compound 12

Figure S20 ATR (FT-IR) spectrum of compound 12

Figure S21 ^1^H-NMR spectrum of compound 13 (DMSO-d_6_, 400 MHz)

Figure S22 ^13^C-NMR spectrum of compound 13 (DMSO-d_6_, 100 MHz)

Figure S23 LC-Q-TOF/MS spectrum of compound 13

Figure S24 ATR (FT-IR) spectrum of compounds 13

Figure S25 ^1^H-NMR spectrum of compound 14 (DMSO-d_6_, 400 MHz)

Figure S26 APT-NMR spectrum of compound 14 (DMSO-d_6_, 100 MHz)

Figure S27 LC-Q-TOF/MS spectrum of compound 14

Figure S28 ATR (FT-IR) spectrum of compound 14

## Figures and Tables

**Figure:**
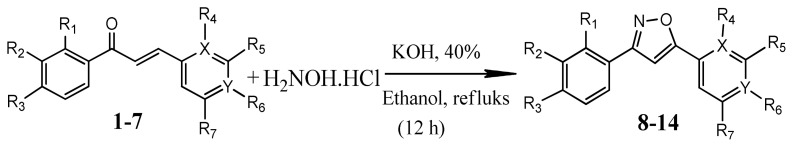
Synthetic scheme for the isoxazole derivatives (**8–14**).

**Table 1:** Tyrosinase inhibition of compounds **8–14**.

No	Tyrosinase (IC_50_ μg/mL)
**8**	66.74 ± 3.96
**9**	No inhibition
**10**	No inhibition
**11**	61.47 ± 3.46
**12**	91.41 ± 4.15
**13**	188.52 ± 5.85
**14**	No inhibition
Kojic acid	3.2 ± 0.29

**Table 2:** Antioxidant (CUPRAC and DPPH) activities of compounds **8–14**.

No	DPPH, SC_50_ (μg/mL)	CUPRAC (mg TEAC/mg)
**8**	61.42 ± 1.41	1.156 ± 0.026
**9**	71.96 ± 2.36	0.856 ± 0.013
**10**	65.65 ± 1.69	0.789 ± 0.009
**11**	81.52 ± 3.66	0.584 ± 0.011
**12**	40.21 ± 2.71	1.233 ± 0.015
**13**	57.32 ± 2.12	1.245 ± 0.019
**14**	54.41 ± 2.87	1.189 ± 0.021
Trolox	5.56 ± 0.85	-
