# Supplementary figures and images for: Synthesis of some new isoxazole compounds and their biological tyrosinase and antioxidant activities
Source: Turk J Chem. 2021 Dec 31;46(3):747–53. doi: 10.55730/1300-0527.3364 (PMC10503991; doi:10.55730/1300-0527.3364)

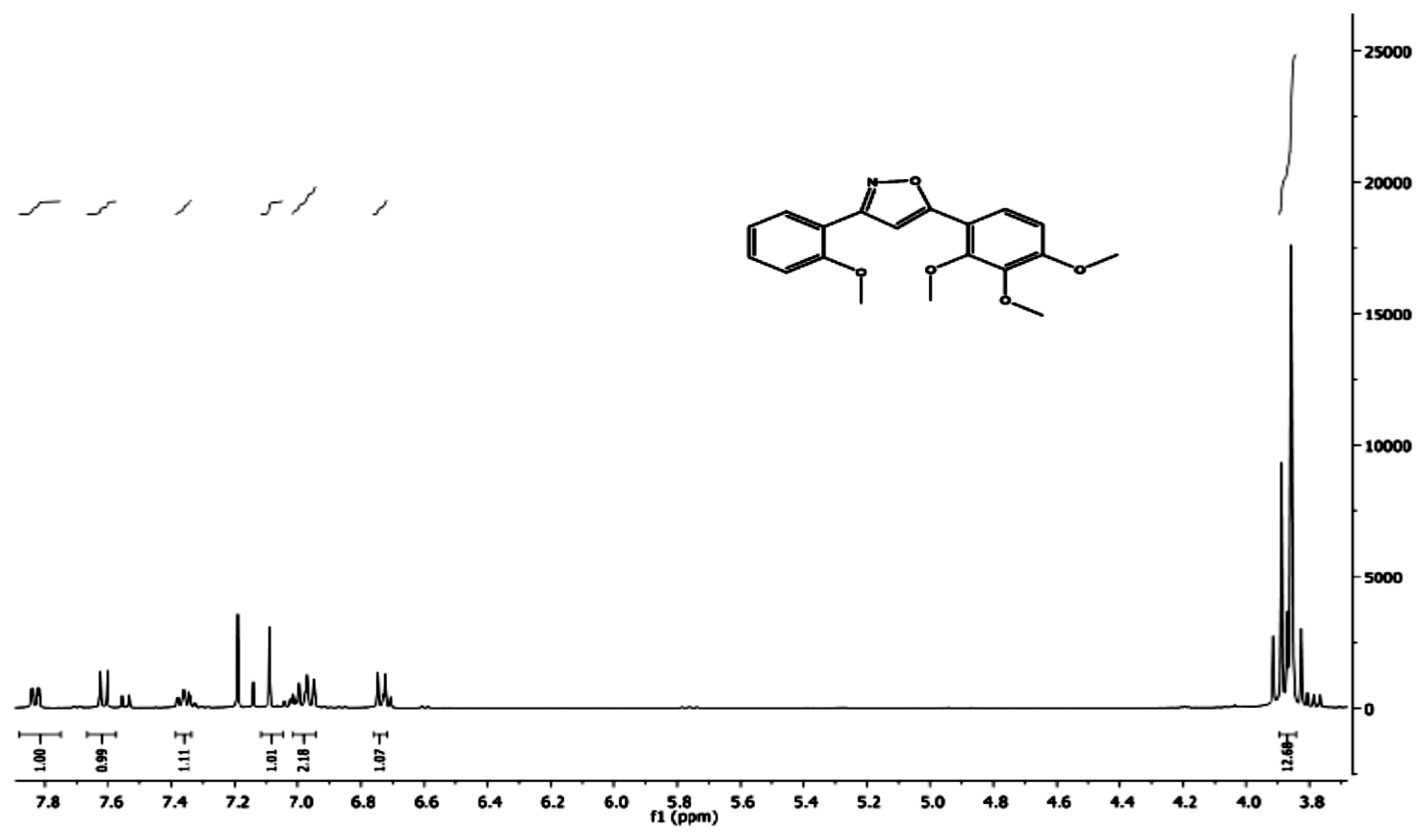

Supplement: Figure S1 — 1H-NMR spectrum of compound 8 (CDCl3, 400 MHz) [file turkjchem-46-3-747s1.tif]

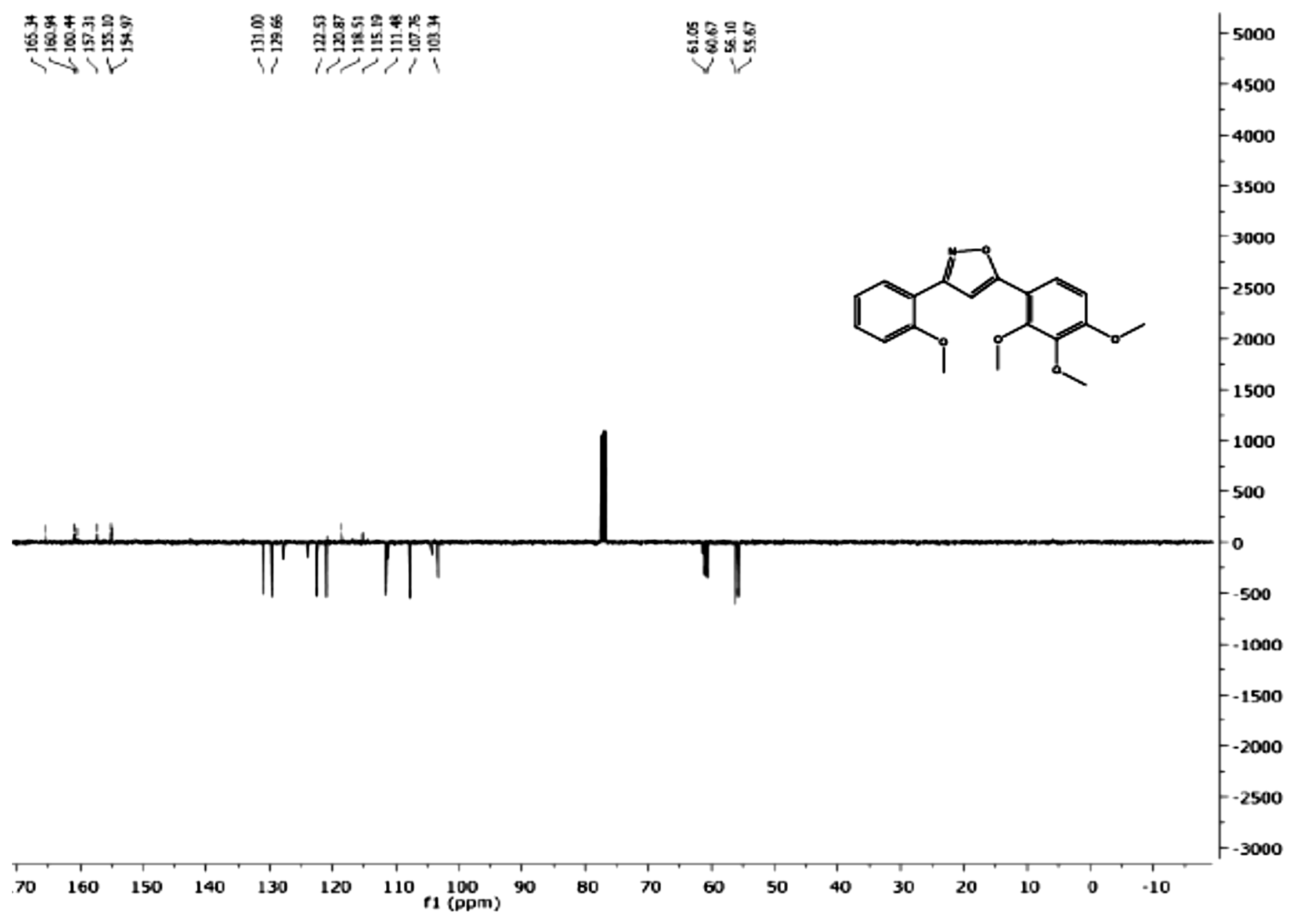

Supplement: Figure S2 — APT-NMR spectrum of compound 8 (CDCl3, 100 MHz) [file turkjchem-46-3-747s2.tif]

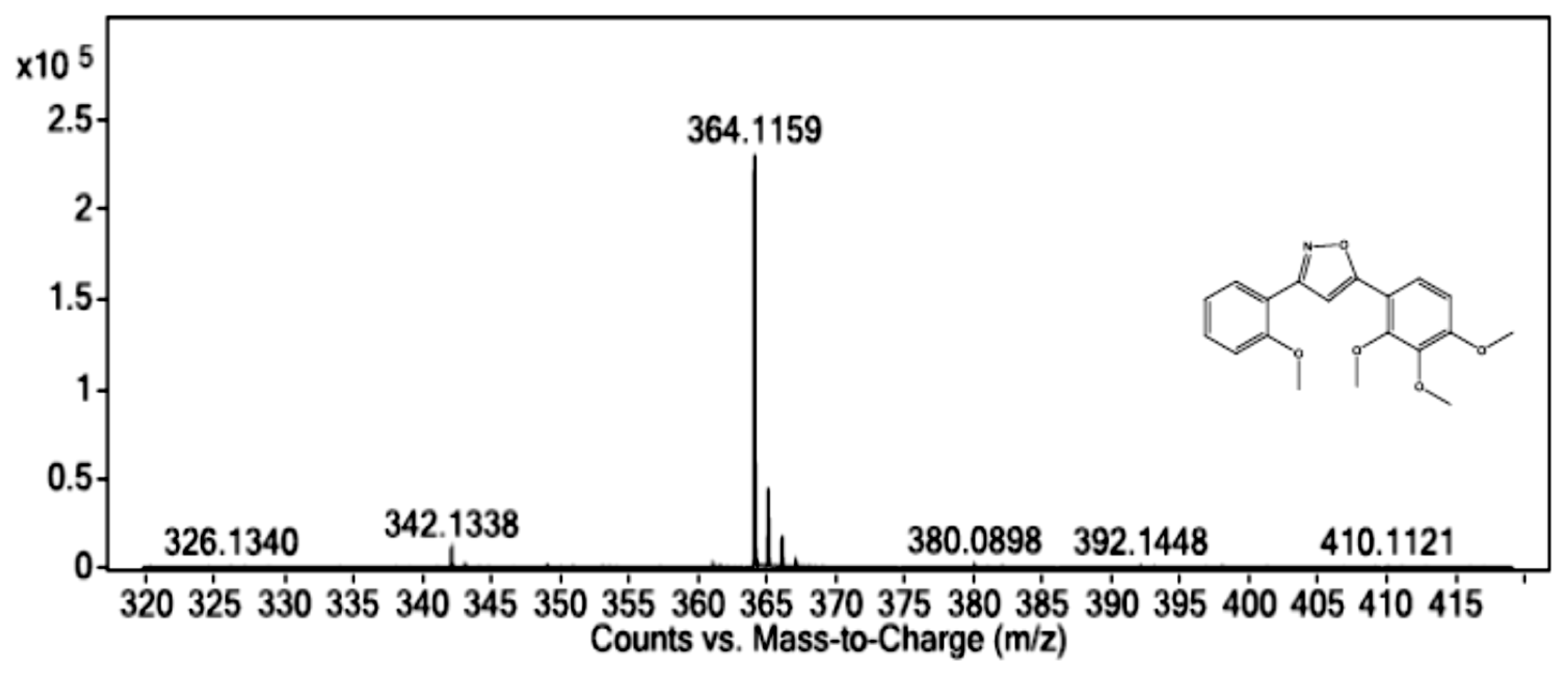

Supplement: Figure S3 — LC-Q-TOF/MS spectrum of compound 8 [file turkjchem-46-3-747s3.tif]

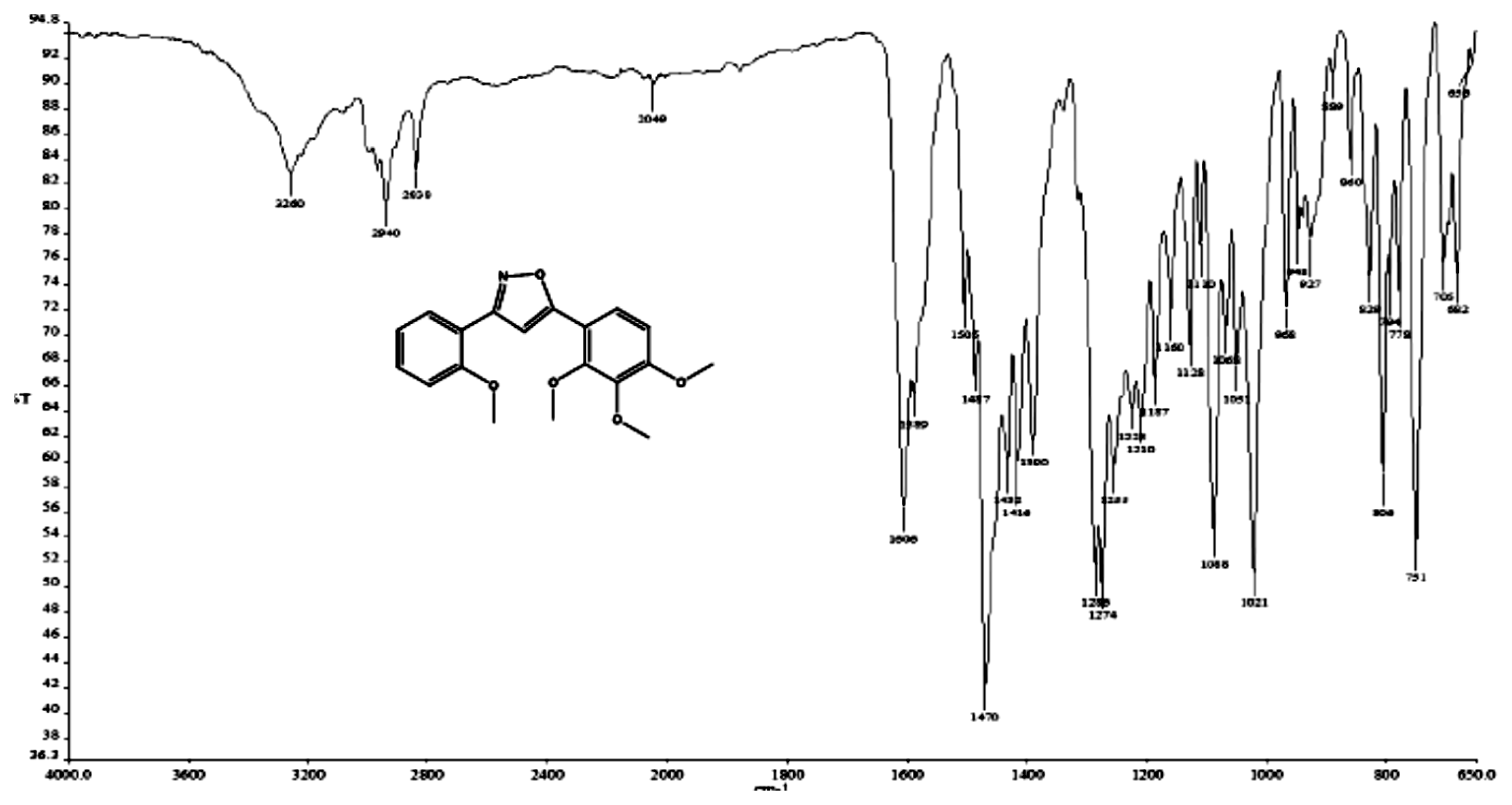

Supplement: Figure S4 — ATR (FT-IR) spectrum of compound 8 [file turkjchem-46-3-747s4.tif]

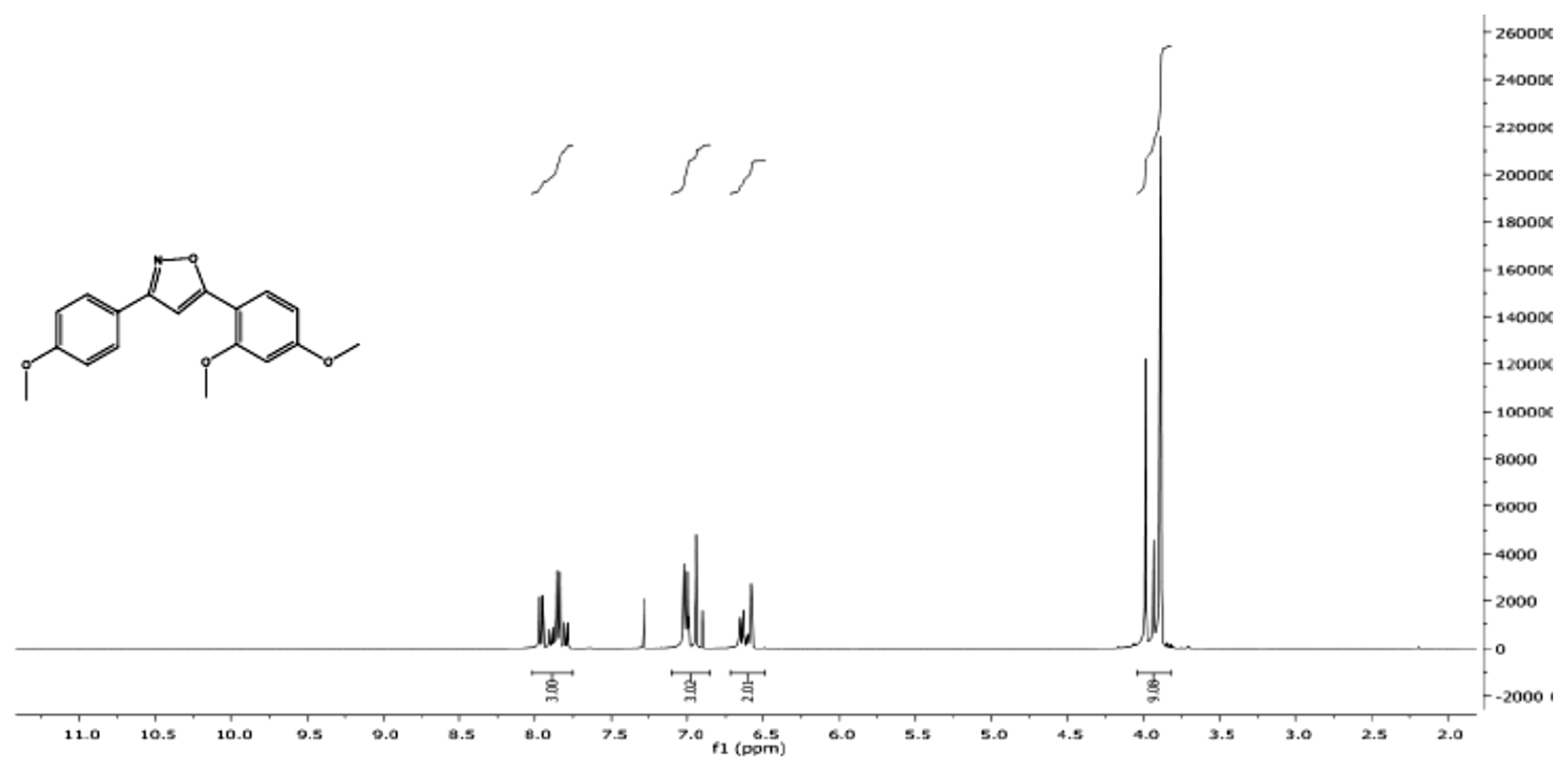

Supplement: Figure S5 — 1H-NMR spectrum of compound 9 (CDCl3, 400 MHz) [file turkjchem-46-3-747s5.tif]

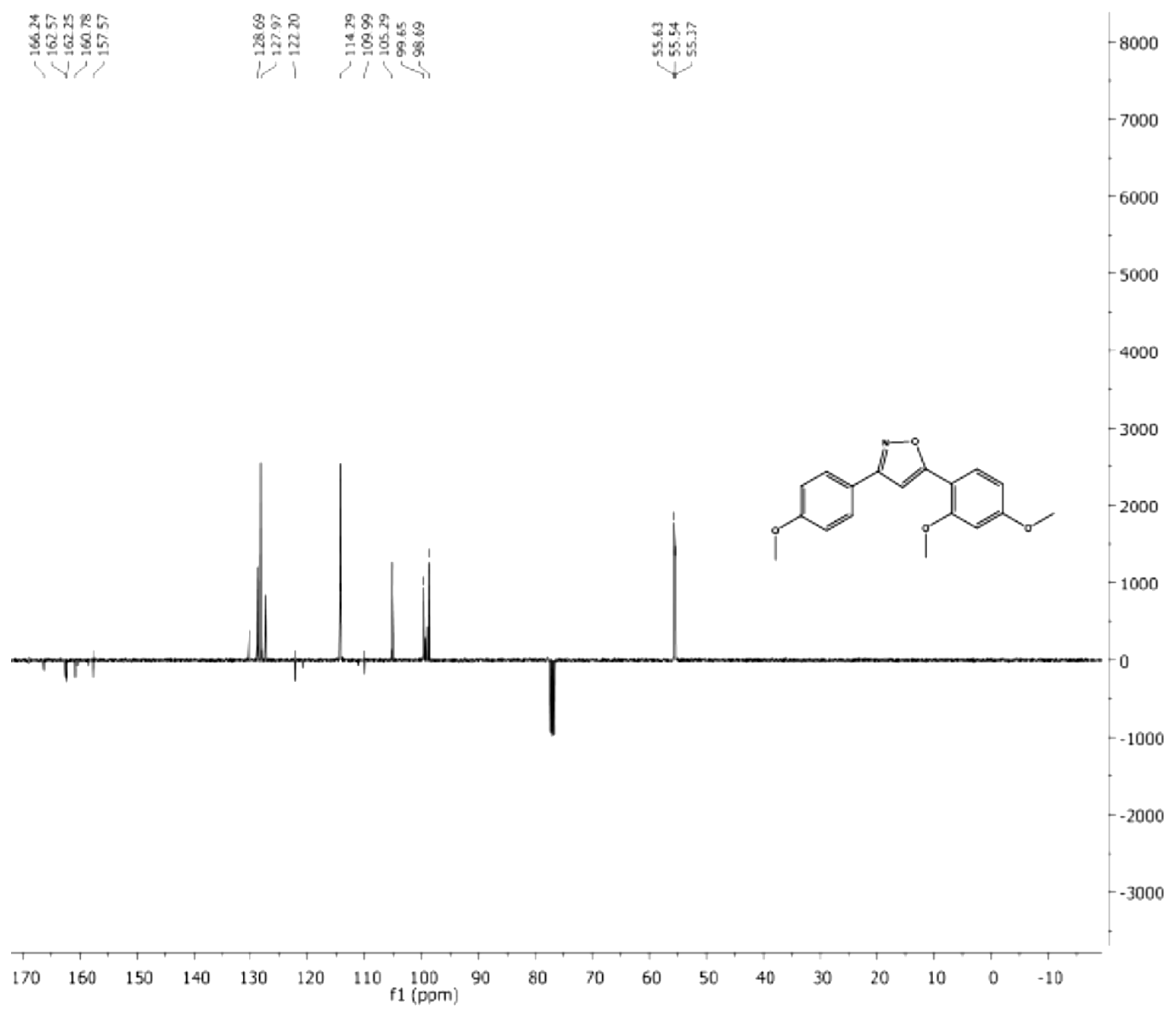

Supplement: Figure S6 — APT-NMR spectrum of compound 9 (CDCl3, 100 MHz) [file turkjchem-46-3-747s6.tif]

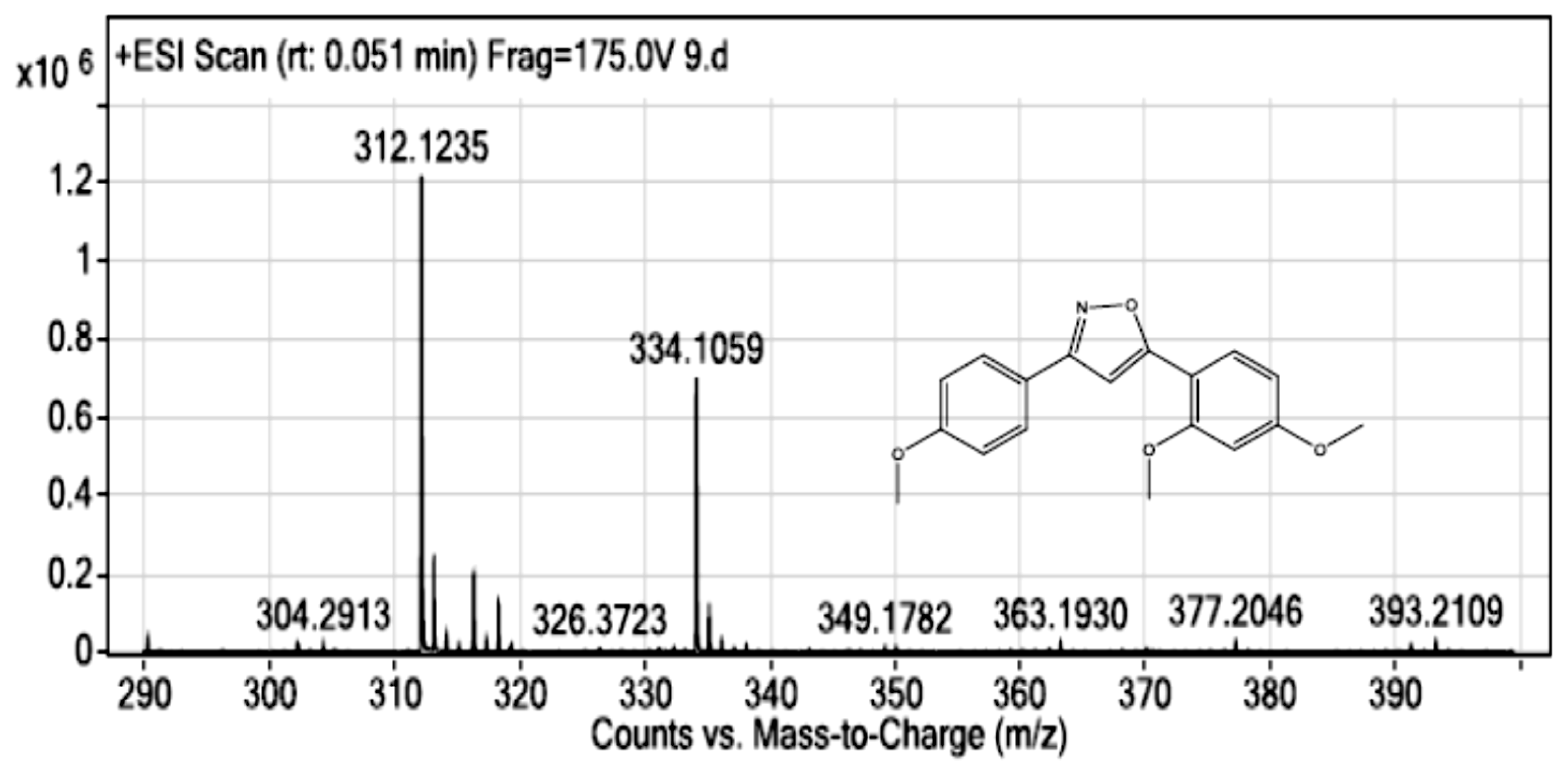

Supplement: Figure S7 — LC-Q-TOF/MS spectrum of compound 9 [file turkjchem-46-3-747s7.tif]

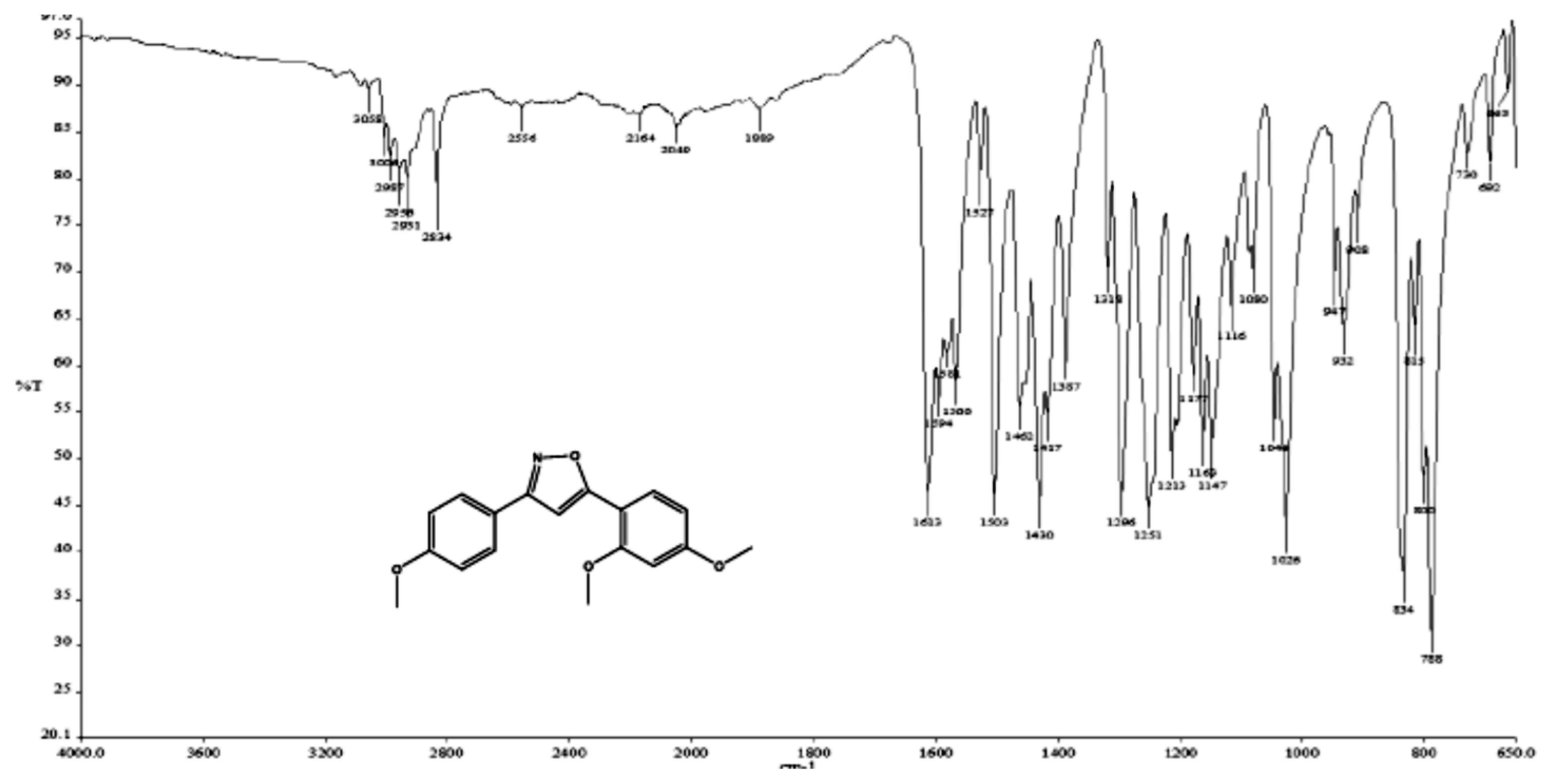

Supplement: Figure S8 — ATR (FT-IR) spektrum of compound 9 [file turkjchem-46-3-747s8.tif]

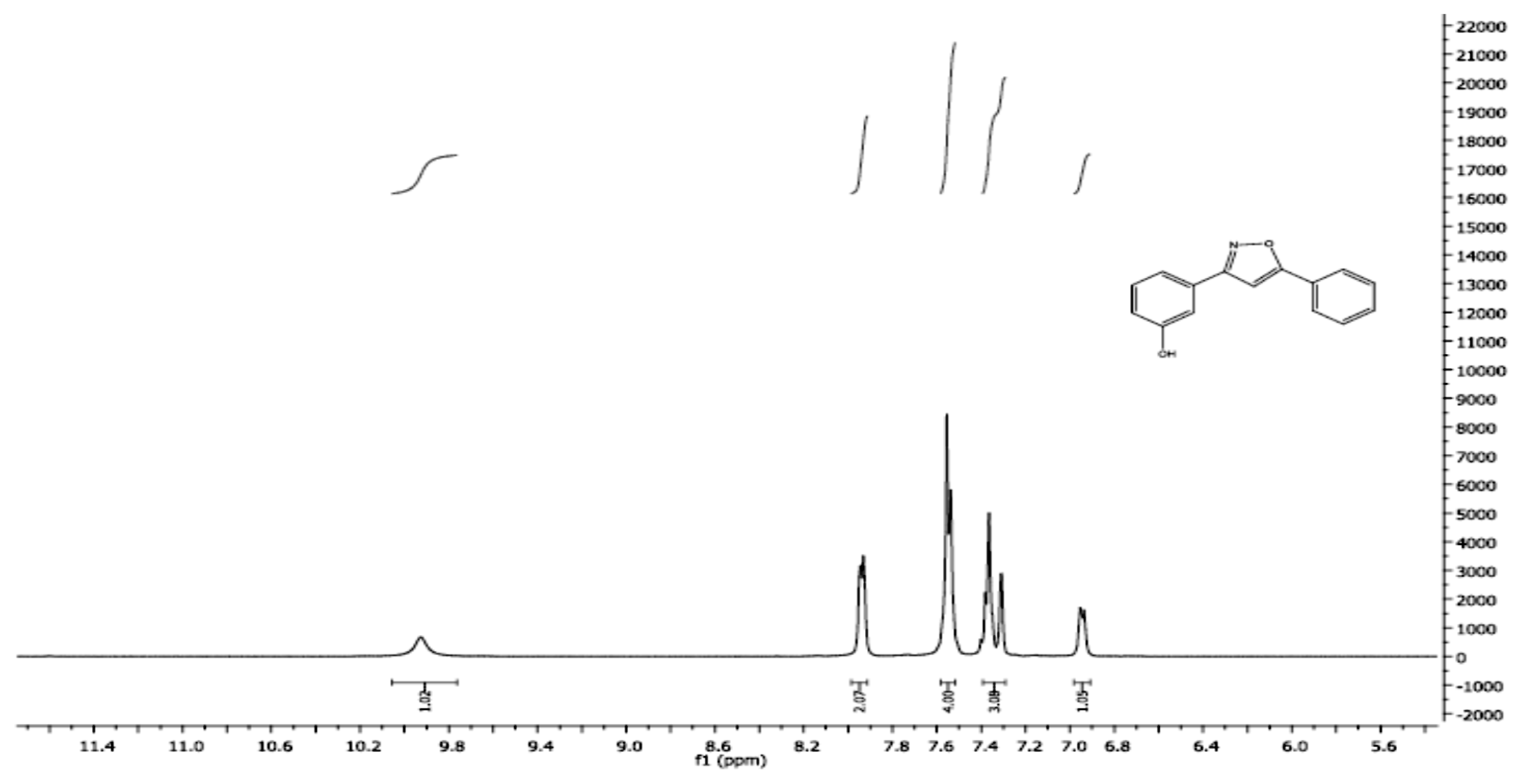

Supplement: Figure S9 — 1H-NMR spectrum of compound 10 (DMSO-d6, 400 MHz) [file turkjchem-46-3-747s9.tif]

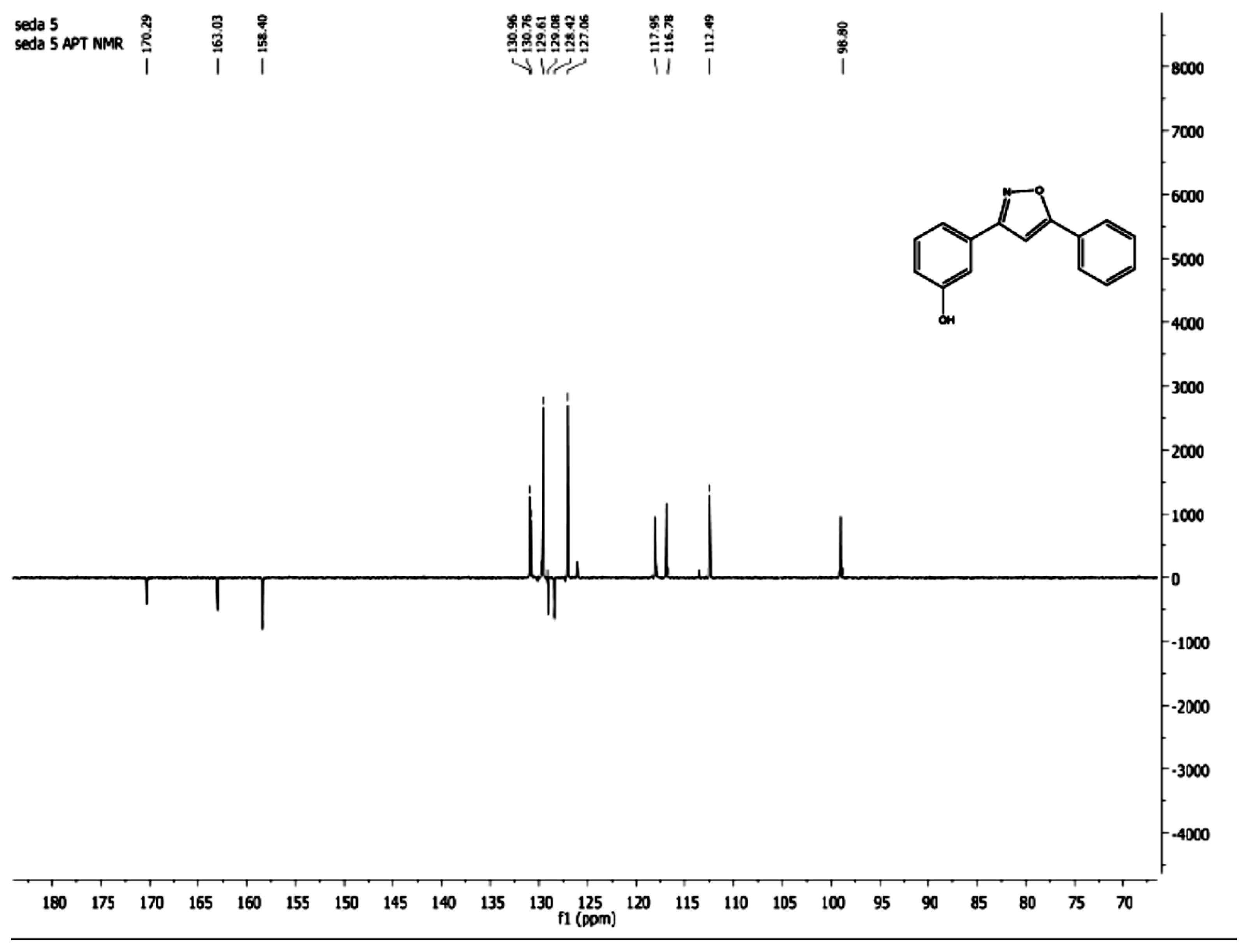

Supplement: Figure S10 — APT-NMR spectrum of compound 10 (DMSO-d6, 100 MHz) [file turkjchem-46-3-747s10.tif]

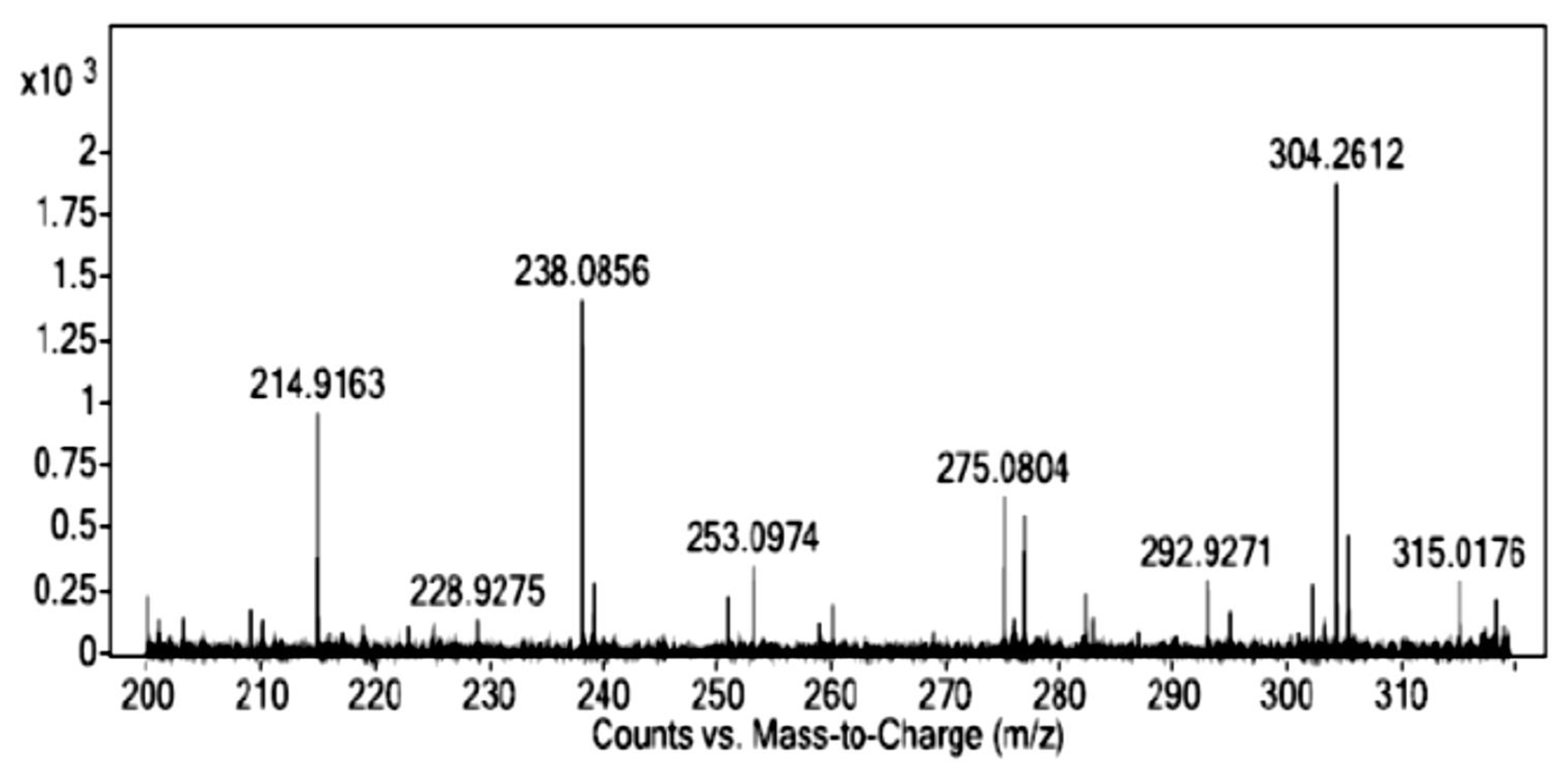

Supplement: Figure S11 — LC-Q-TOF/MS spectrum of compound 10 [file turkjchem-46-3-747s11.tif]

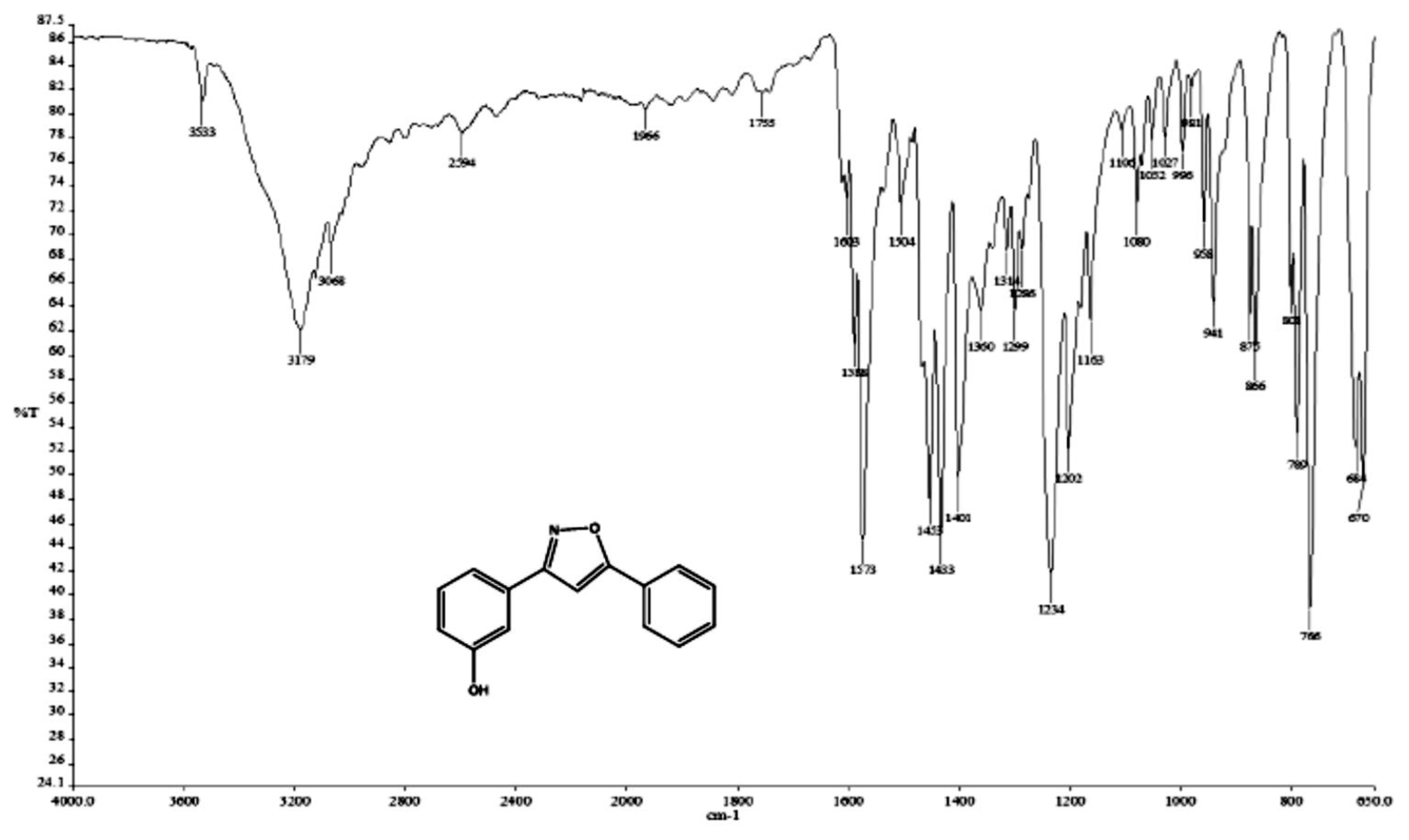

Supplement: Figure S12 — ATR (FT-IR) spektrum of compound 10 [file turkjchem-46-3-747s12.tif]

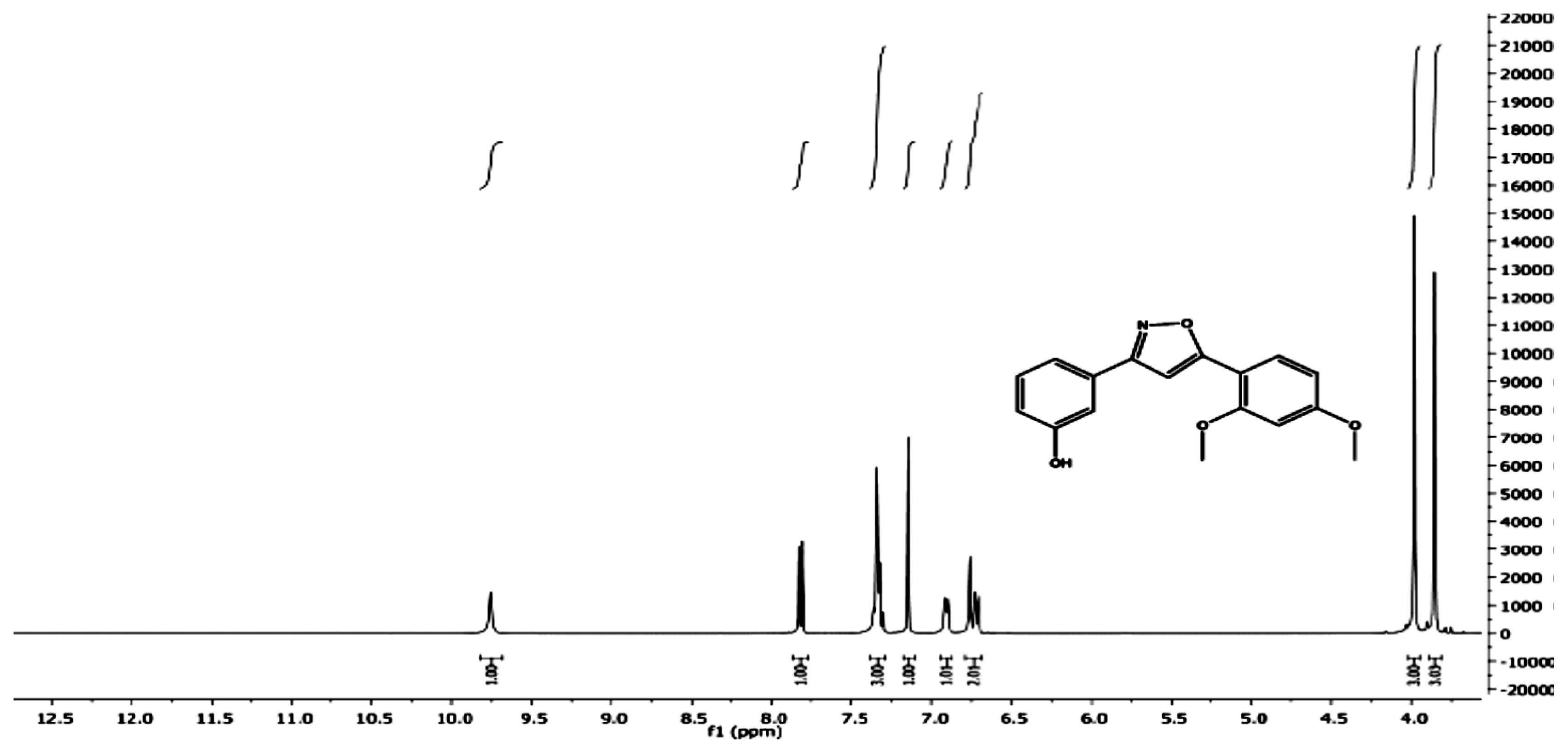

Supplement: Figure S13 — 1H-NMR spectrum of compound 11 (DMSO-d6, 400 MHz) [file turkjchem-46-3-747s13.tif]

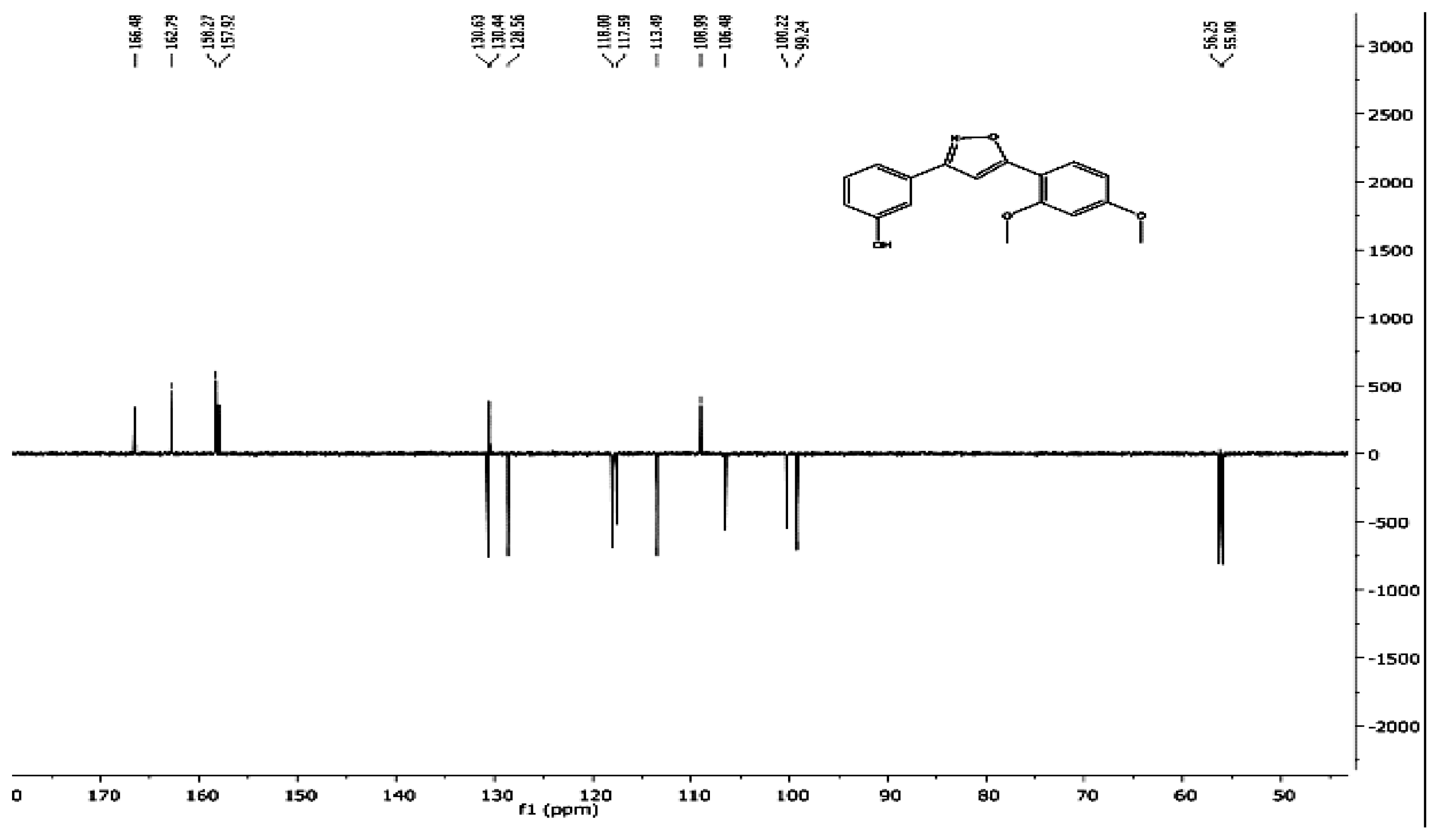

Supplement: Figure S14 — APT-NMR spectrum of compound 11 (DMSO-d6, 100 MHz) [file turkjchem-46-3-747s14.tif]

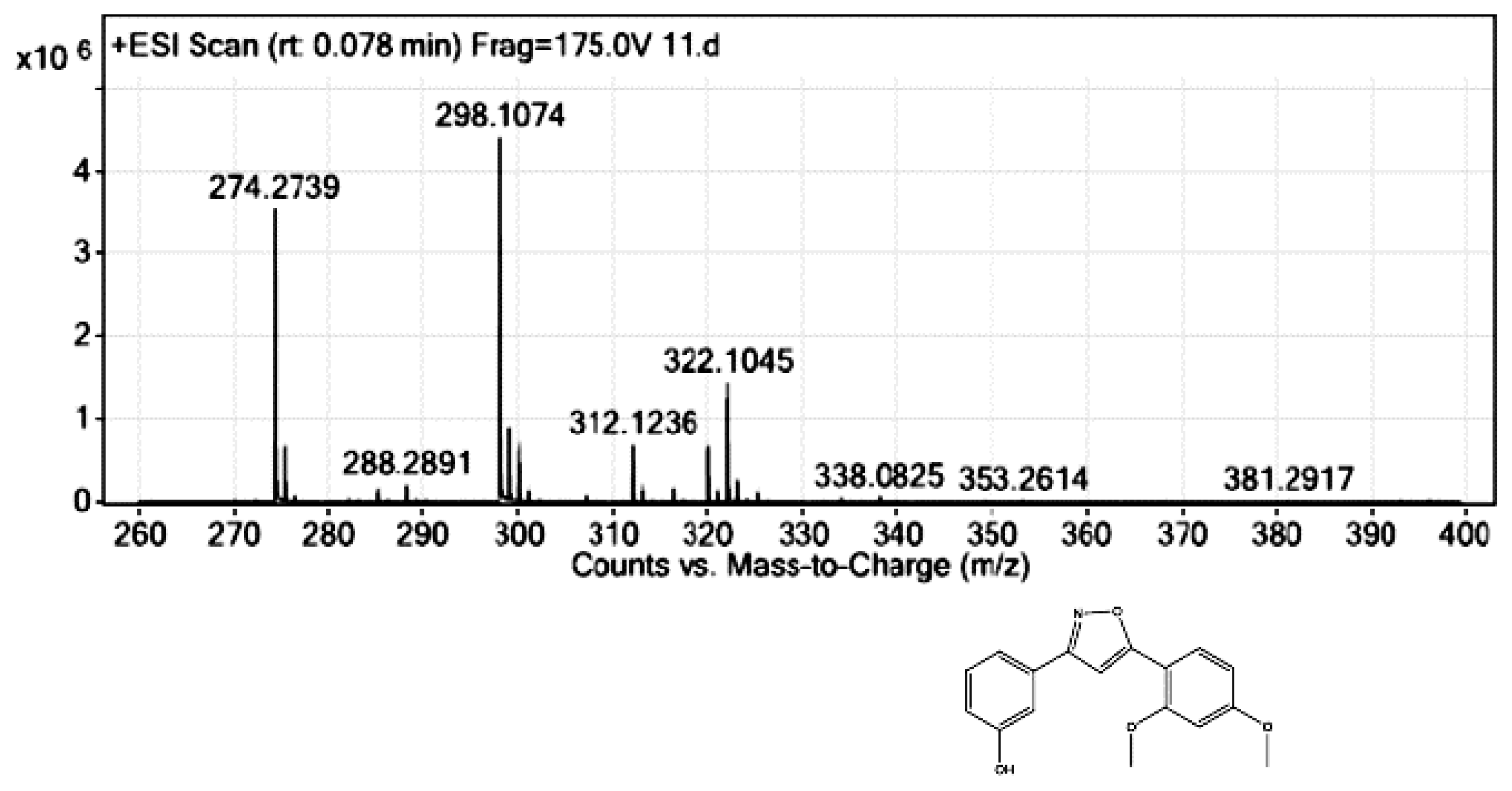

Supplement: Figure S15 — LC-Q-TOF/MS spectrum of compound 11 [file turkjchem-46-3-747s15.tif]

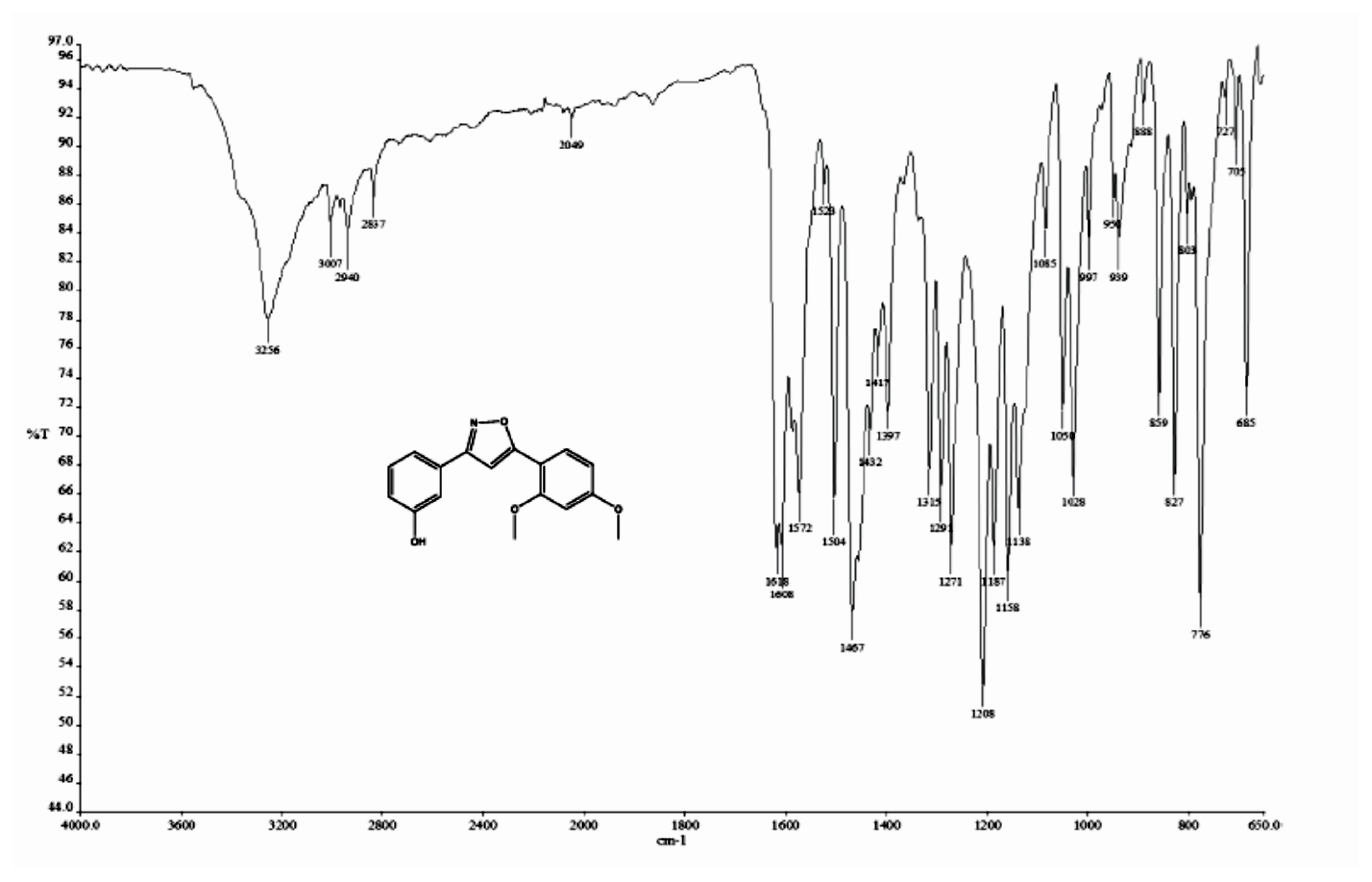

Supplement: Figure S16 — ATR (FT-IR) spectrum of compound 11 [file turkjchem-46-3-747s16.tif]

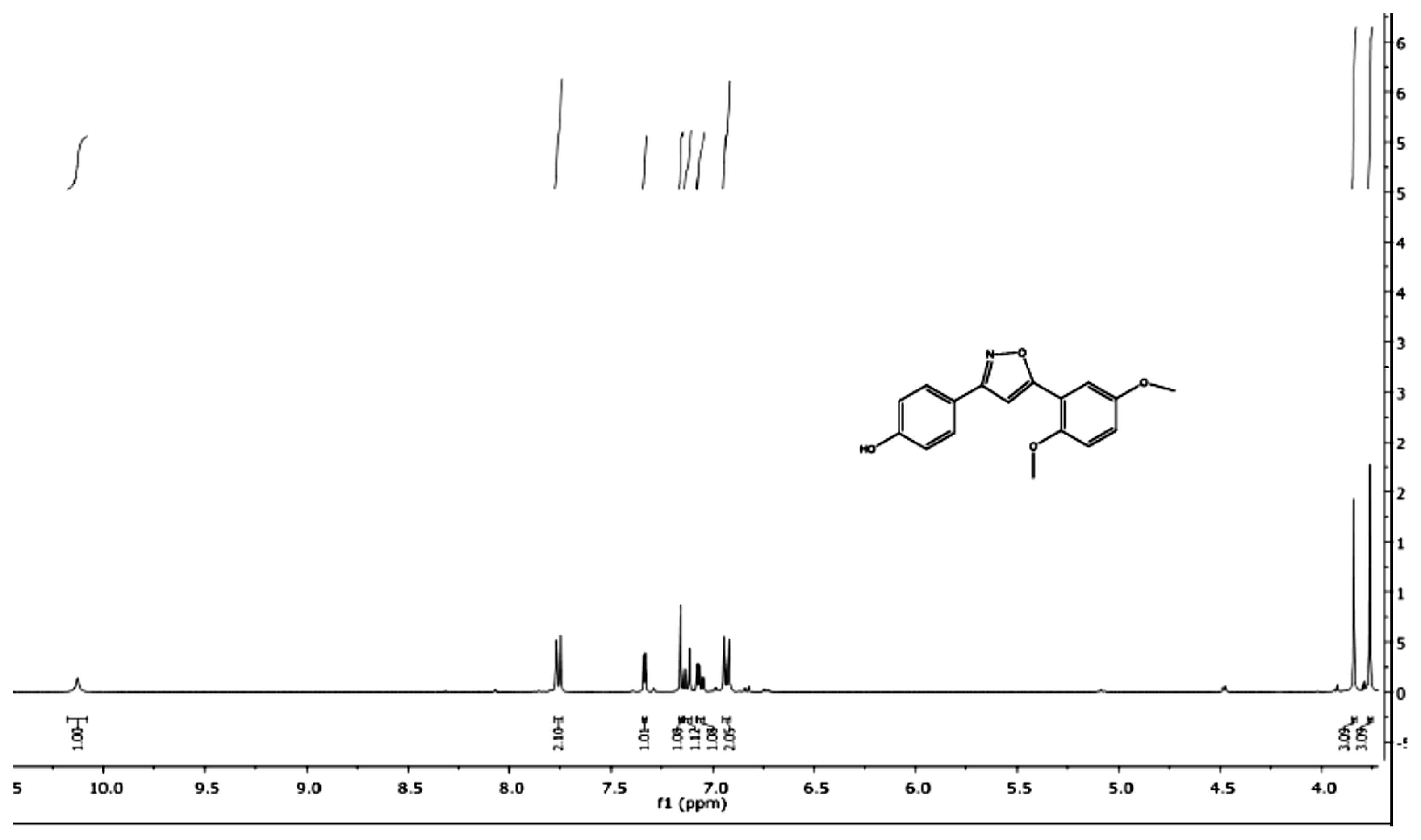

Supplement: Figure S17 — 1H-NMR spectrum of compound 12 (DMSO-d6, 400 MHz) [file turkjchem-46-3-747s17.tif]

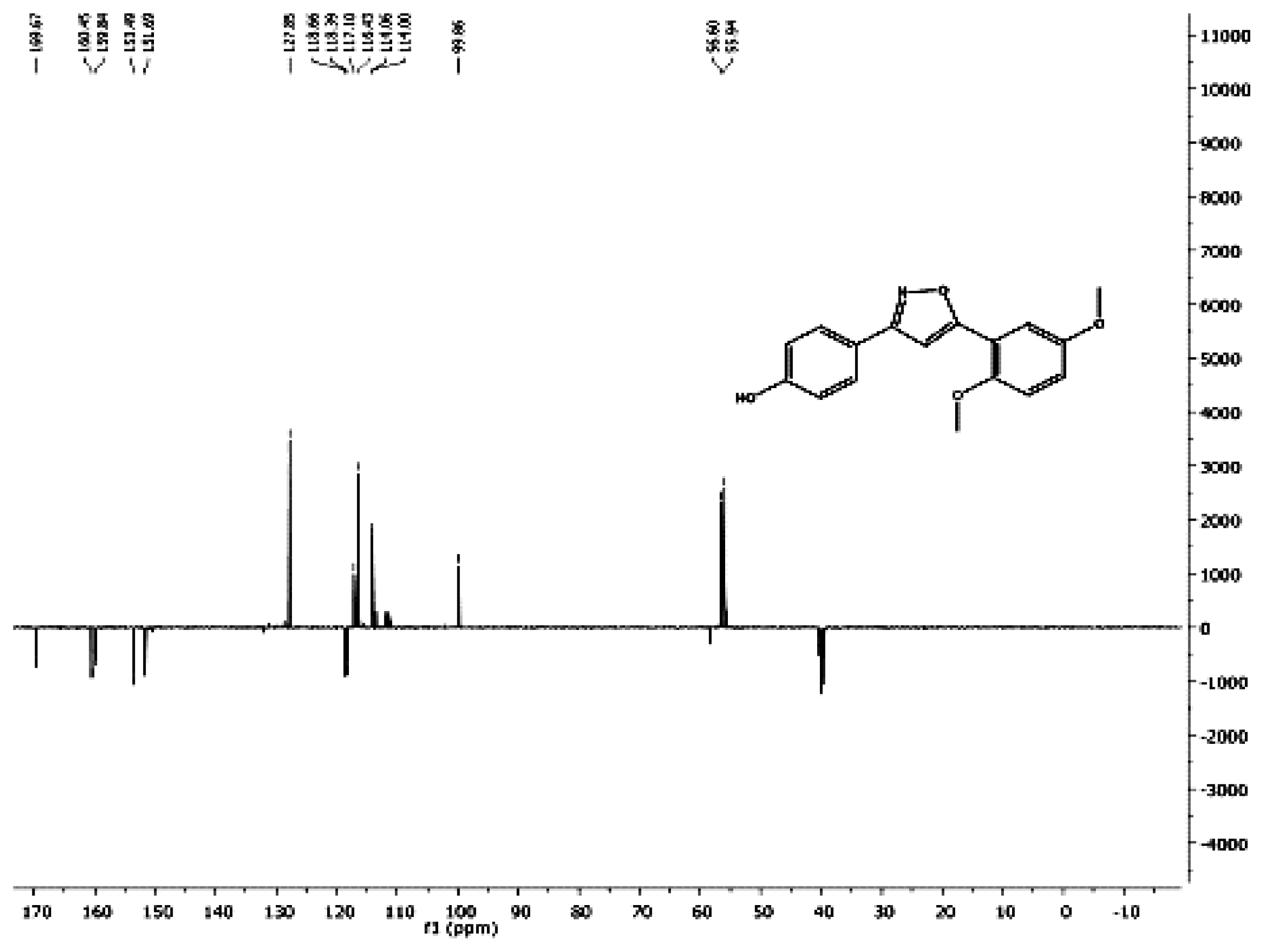

Supplement: Figure S18 — APT-NMR spectrum of compound 12 (DMSO-d6, 100 MHz) [file turkjchem-46-3-747s18.tif]

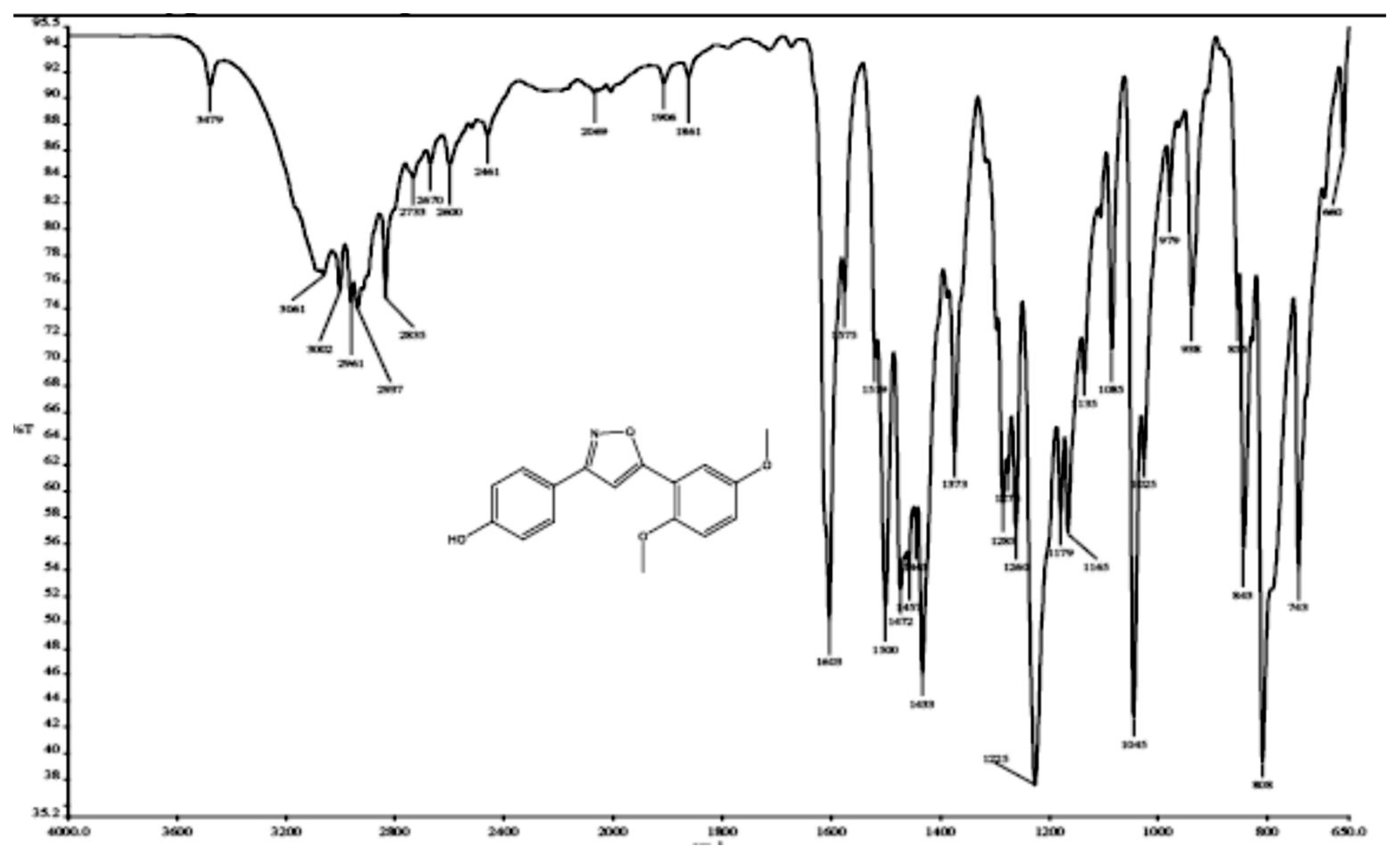

Supplement: Figure S19 — LC-Q-TOF/MS spectrum of compound 12 [file turkjchem-46-3-747s19.tif]

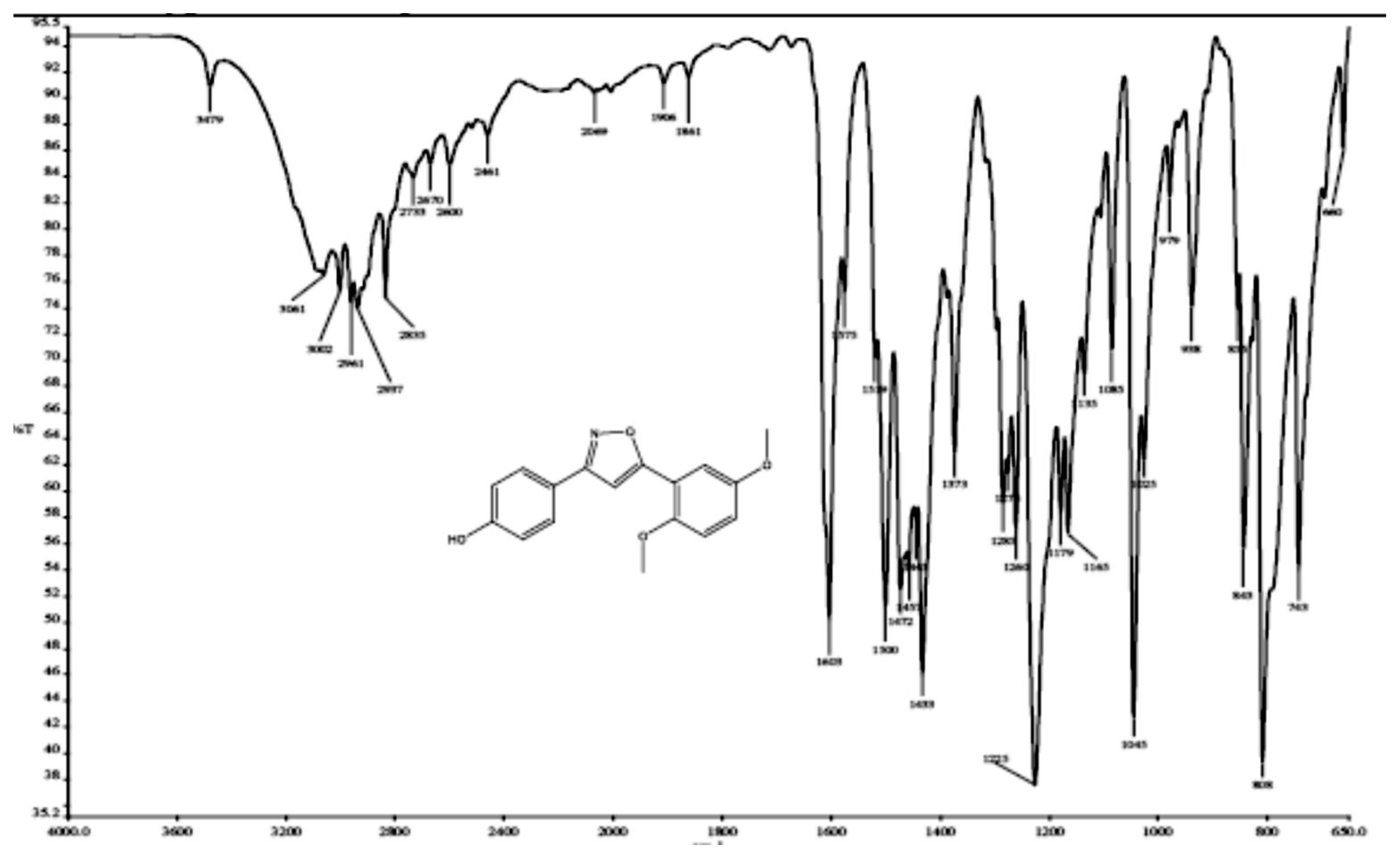

Supplement: Figure S20 — ATR (FT-IR) spectrum of compound 12 [file turkjchem-46-3-747s20.tif]

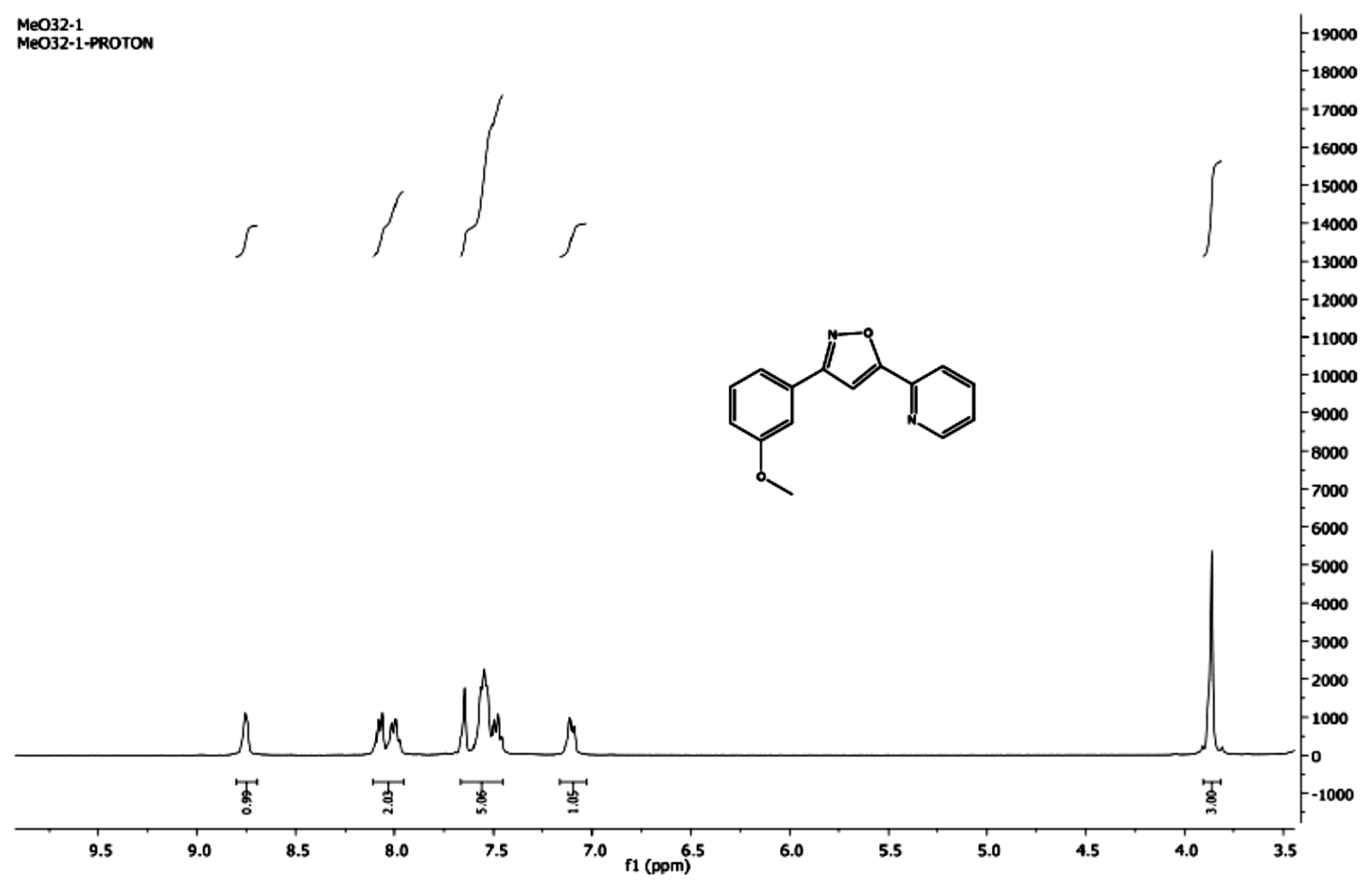

Supplement: Figure S21 — 1H-NMR spectrum of compound 13 (DMSO-d6, 400 MHz) [file turkjchem-46-3-747s21.tif]

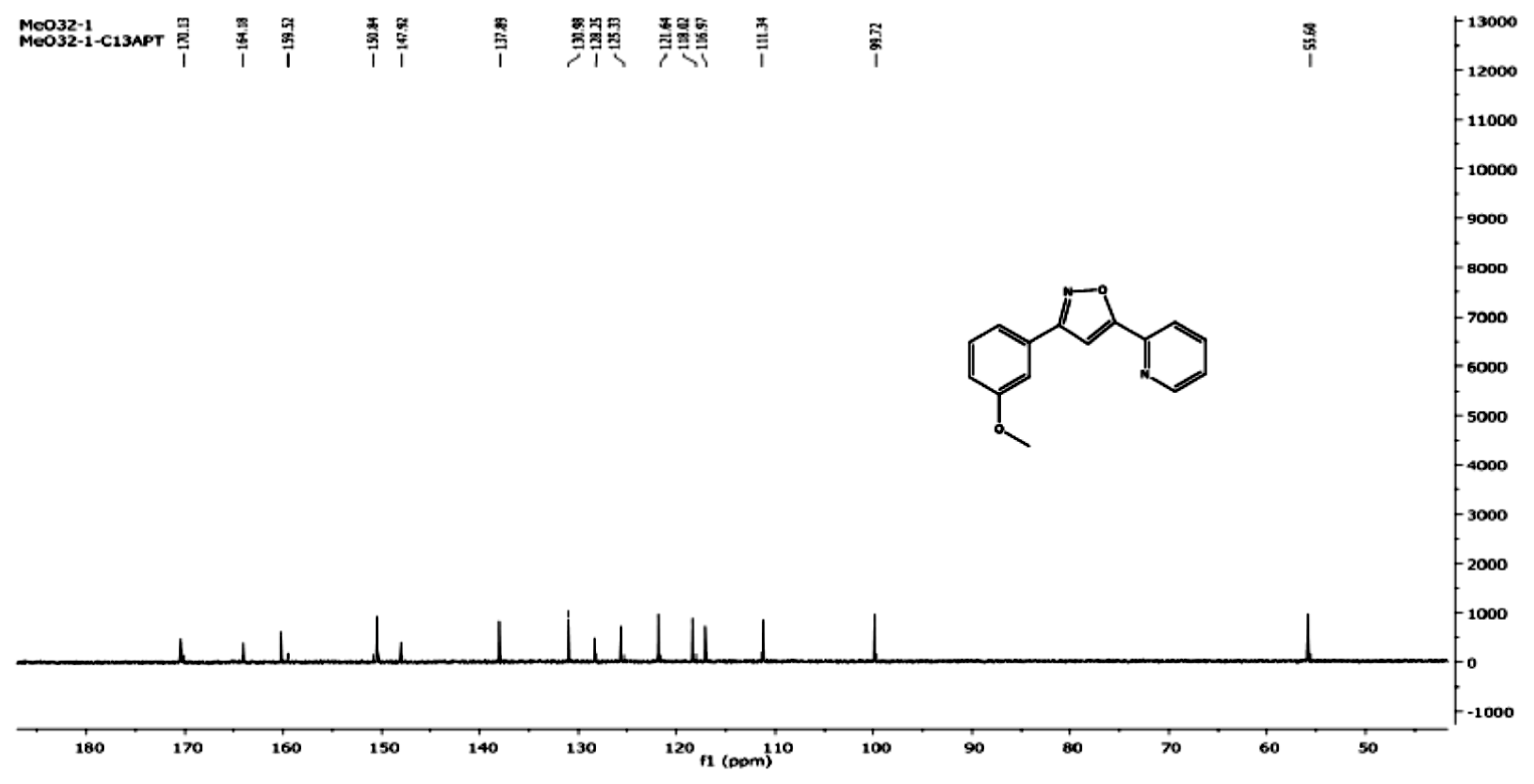

Supplement: Figure S22 — 13C-NMR spectrum of compound 13 (DMSO-d6, 100 MHz) [file turkjchem-46-3-747s22.tif]

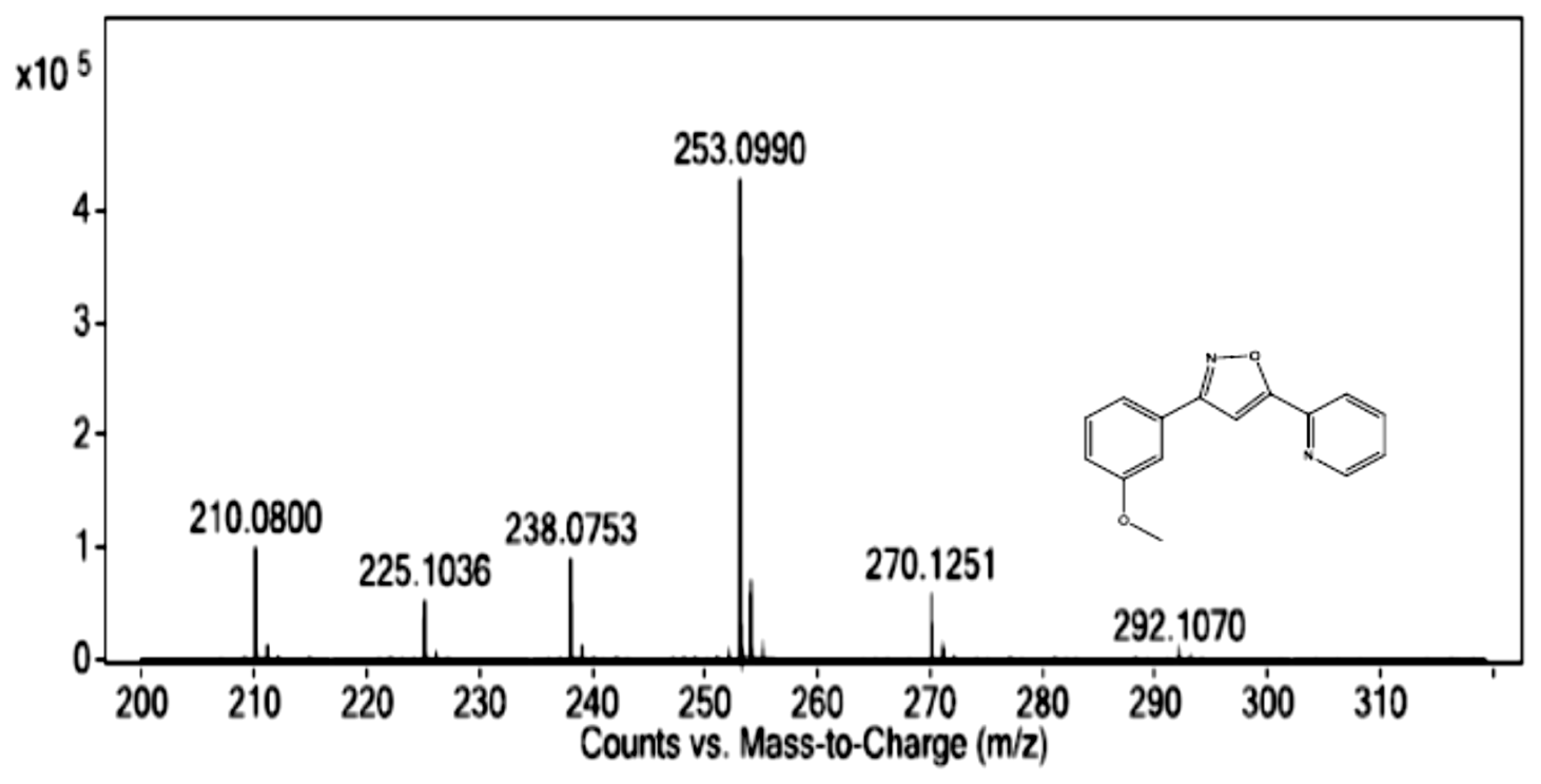

Supplement: Figure S23 — LC-Q-TOF/MS spectrum of compound 13 [file turkjchem-46-3-747s23.tif]

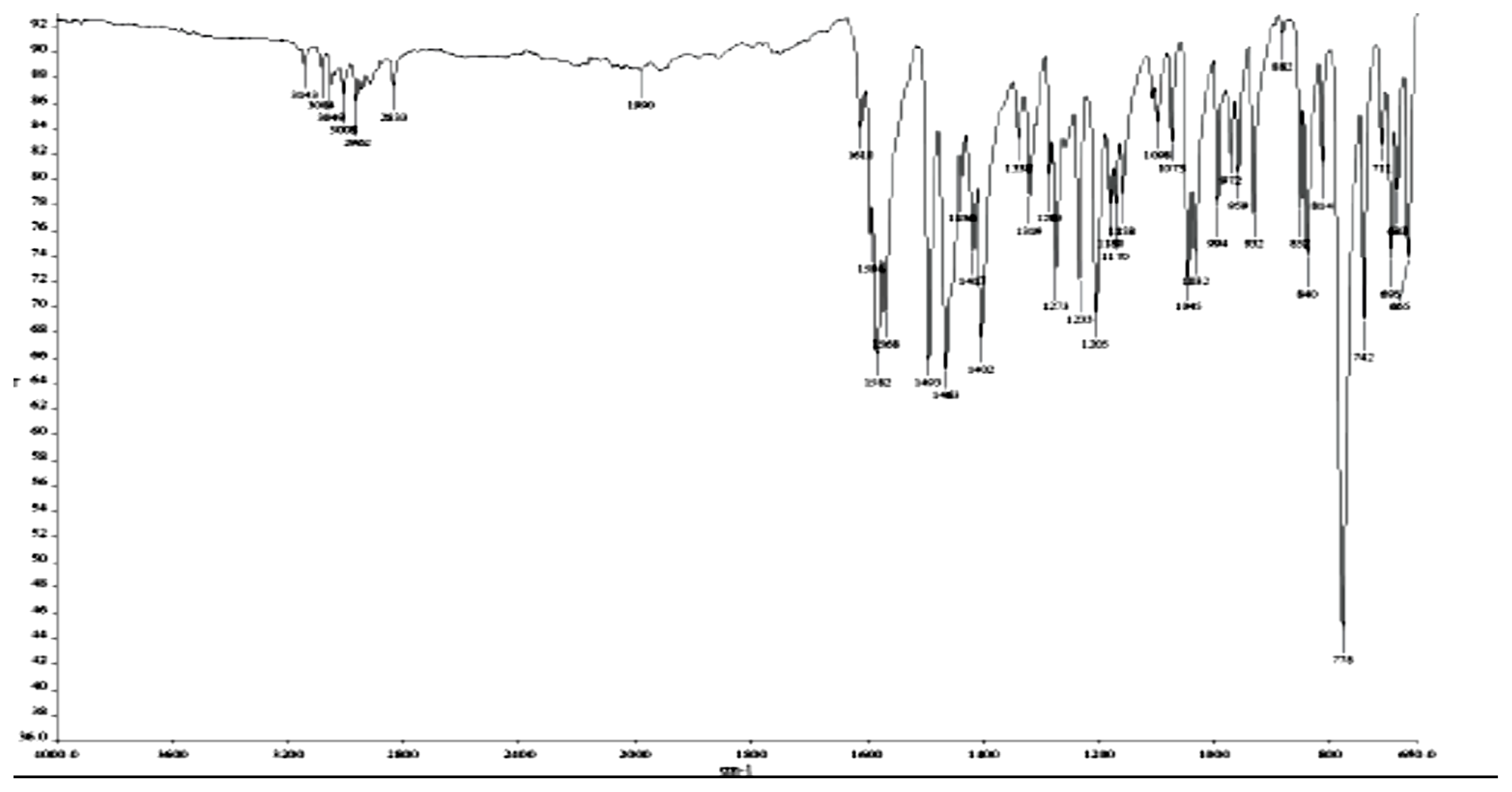

Supplement: Figure S24 — ATR (FT-IR) spectrum of compounds 13 [file turkjchem-46-3-747s24.tif]

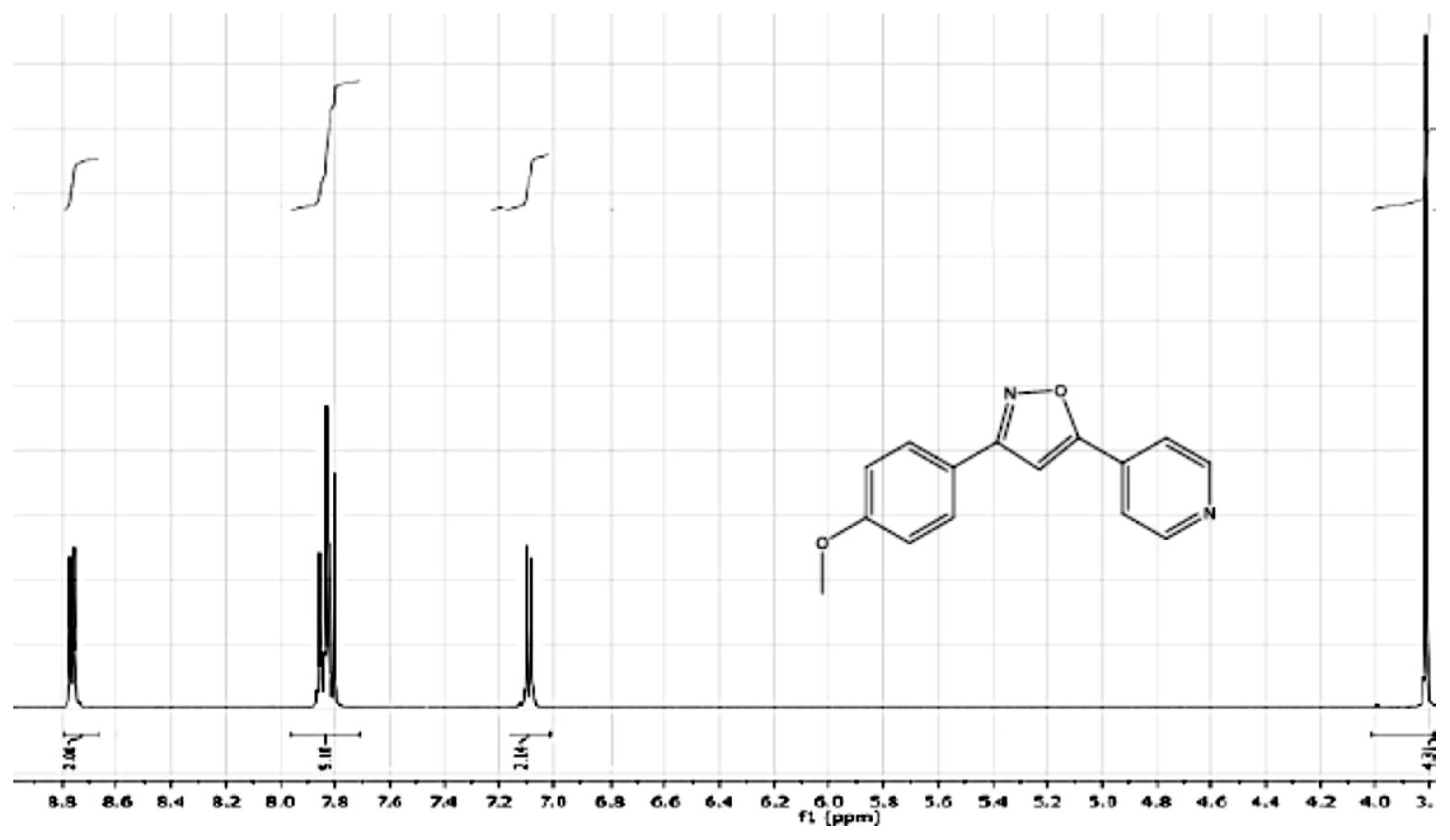

Supplement: Figure S25 — 1H-NMR spectrum of compound 14 (DMSO-d6, 400 MHz) [file turkjchem-46-3-747s25.tif]

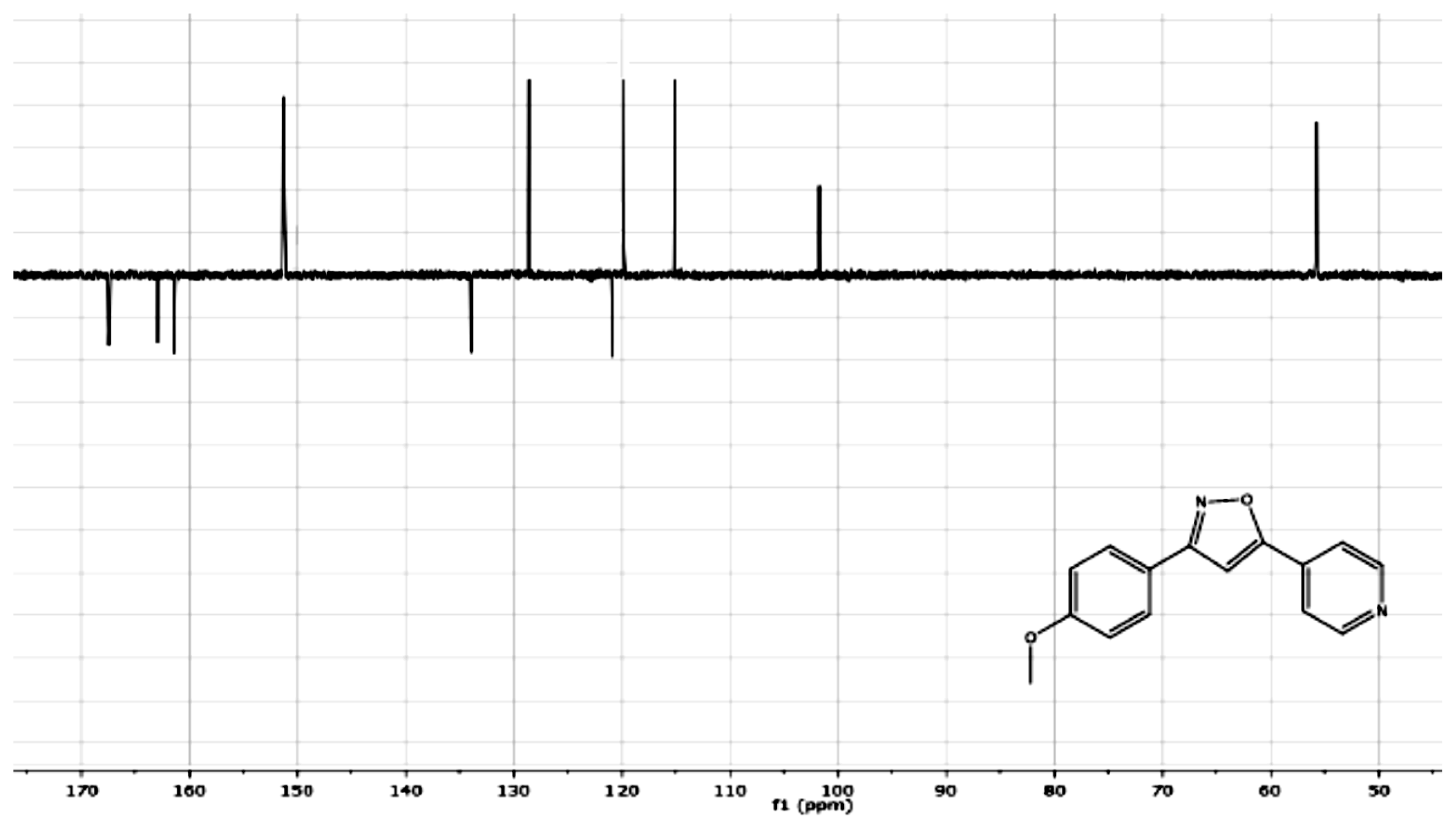

Supplement: Figure S26 — APT-NMR spectrum of compound 14 (DMSO-d6, 100 MHz) [file turkjchem-46-3-747s26.tif]

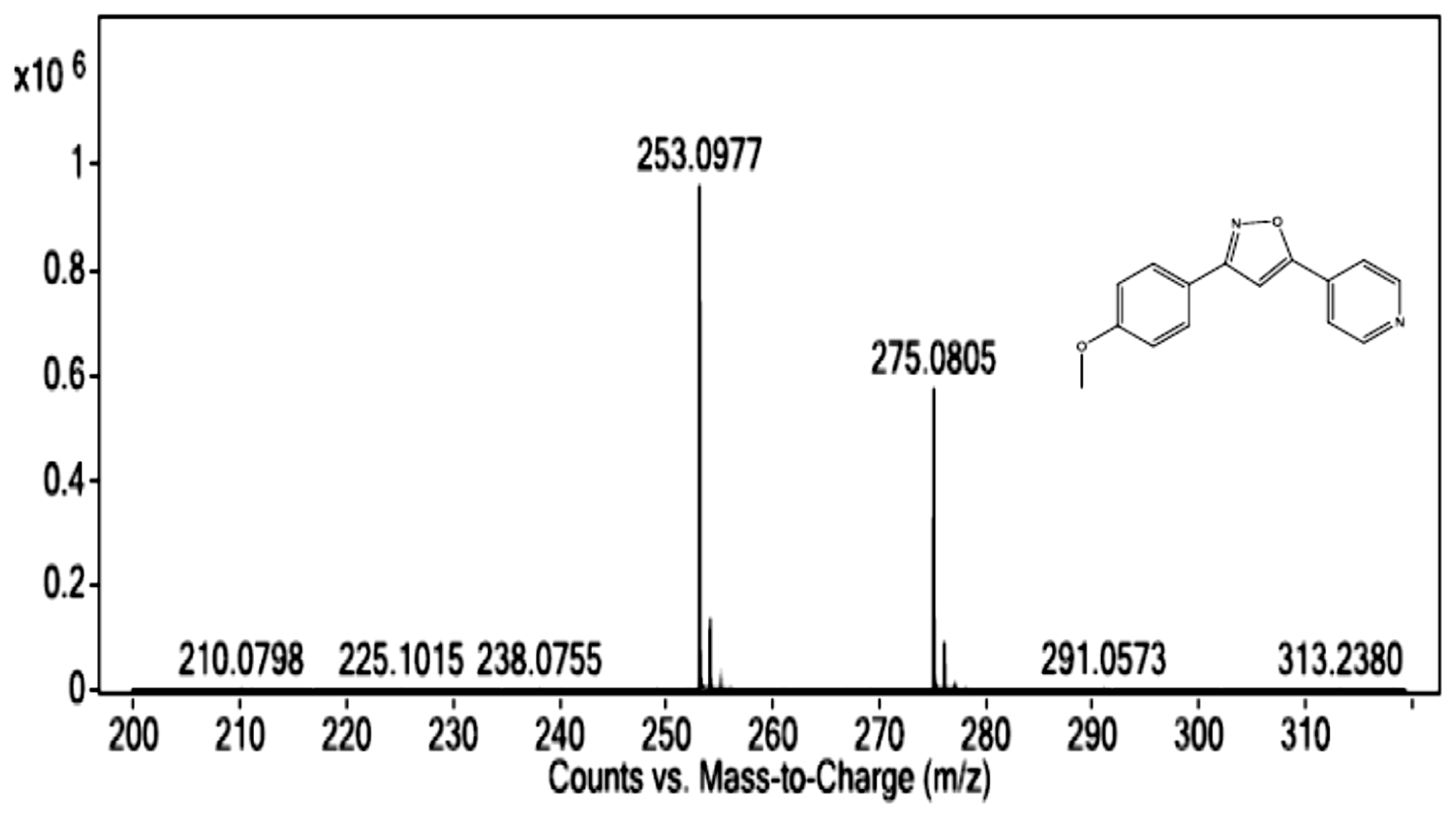

Supplement: Figure S27 — LC-Q-TOF/MS spectrum of compound 14 [file turkjchem-46-3-747s27.tif]

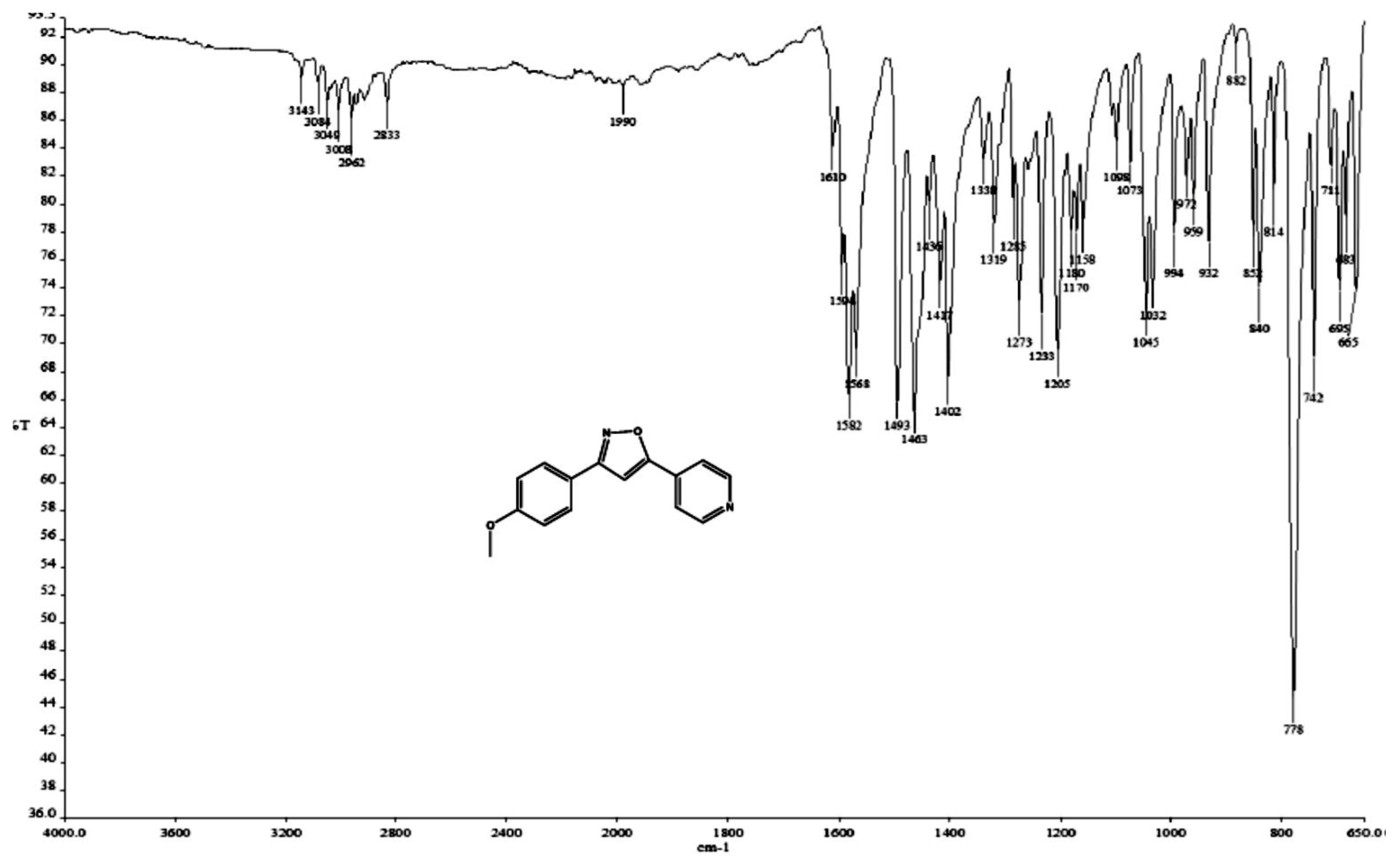

Supplement: Figure S28 — ATR (FT-IR) spectrum of compound 14 [file turkjchem-46-3-747s28.tif]
